# Analysis of genotype × environment interactions for agronomic traits of soybean (*Glycine max* [L.] Merr.) using association mapping

**DOI:** 10.3389/fgene.2022.1090994

**Published:** 2023-01-05

**Authors:** Reena Rani, Ghulam Raza, Hamza Ashfaq, Muhammad Rizwan, Hussein Shimelis, Muhammad Haseeb Tung, Muhammad Arif

**Affiliations:** ^1^ DNA Markers and Applied Genomics Lab, Agricultural Biotechnology Division, National Institute for Biotechnology and Genetic Engineering (NIBGE), Constituent College Pakistan Institute of Engineering and Applied Sciences (PIEAS), Faisalabad, Pakistan; ^2^ Plant Breeding and Genetics Division, Nuclear Institute of Agriculture (NIA), Tando Jam, Pakistan; ^3^ School of Agricultural, Earth and Environmental Sciences, African Centre for Crop Improvement, University of KwaZulu-Natal, Pietermaritzburg, South Africa

**Keywords:** genotype-by-environment interaction, association mapping, GGE biplots, agronomic traits, soybean

## Abstract

The soybean yield is a complex quantitative trait that is significantly influenced by environmental factors. G × E interaction (GEI), which derives the performance of soybean genotypes differentially in various environmental conditions, is one of the main obstacles to increasing the net production. The primary goal of this study is to identify the outperforming genotypes in different latitudes, which can then be used in future breeding programs. A total of 96 soybean genotypes were examined in two different ecological regions: Faisalabad and Tando Jam in Pakistan. The evaluation of genotypes in different environmental conditions showed a substantial amount of genetic diversity for grain yield. We identified 13 environment-specific genotypes showing their maximum grain yield in each environment. Genotype G69 was found to be an ideal genotype with higher grain yield than other genotypes tested in this study and is broadly adapted for environments E1 and E2 and also included in top-yielding genotypes in E3, E4, and E5. G92 is another genotype that is broadly adapted in E1, E3, and E4. In the case of environments, E3 is suggested to be a more ideal environment as it is plotted near the concentric circle and is very informative for the selection of genotypes with high yield. Despite the presence of GEI, advances in DNA technology provided very useful tools to investigate the insight of advanced genotypes. Association mapping is a useful method for swiftly and efficiently investigating the genetic basis of significant plant traits. A total of 26 marker–trait associations were found for six agronomic traits in five environments, with the highest significance (*p*-value = 2.48 × 10^–08^) for plant height and the lowest significance (1.03 × 10^–03^) for hundred-grain weight. Soybean genotypes identified in the present study could be a valuable source for future breeding programs as they are adaptable to a wide range of environments. Genetic selection of genotypes with the best yields can be used for gross grain production in a wide range of climatic conditions, and it would give an essential reference in terms of soybean variety selection.

## 1 Introduction

The soybean (*Glycine max*) is an important oil seed crop that fulfills the demands of oil and proteins of millions of people around the world. Its cultivation area covered 130.94 million hectares during 2021/2022, with a net production of 355.59 million metric tons in the world (https://www.fas.usda.gov/data/world-agricultural-production). However, in Pakistan, its cultivation area is negligible, with a production of 1,000 metric tons. To meet the increasing need for food, it is essential for breeders to develop cultivars that have high yield and yield stability and are also resistant to biotic and abiotic stresses (Eltaher et al., 2021). In any crop species, grain yield is the most important factor. It is a complex quantitative trait that is influenced by multiple genes and environmental factors (Yongchun et al., 2008). Hence, it is necessary to dissect the underlying genetics of grain yield and other related traits for manipulating alleles at relevant loci to get maximum benefits (Yongchun et al., 2008). The selection of genotypes carried out in a single environment on the basis of their performance is not suitable for the development of varieties (Shrestha et al., 2012). So, the selection of the genotypes on the basis of yield stability evaluation is more important than their mean performance in multiple environmental conditions (Tariku et al., 2013; Islam et al., 2016). For crops like soybean, which grows in a wide range of ecological conditions, it is very important to select the genotypes for adaptability and stability before recommending any environmental condition. The photoperiod is the main climatic factor in soybean that determines its adaptability to different ecological conditions. Because of photoperiod sensitivity, each soybean cultivar is restricted to cultivation in a narrow range of latitudes to get maximum yield (Debebe et al., 2014). Although soybean grows in a wide range of latitudes (50°N–35°S) across the world, identification of traits that help to determine the performance of the most stable cultivars at different latitudes is very important (Li et al., 2020).

Genotype × environment interaction (GEI) has limitations in the study of important agronomic traits like yield and its components, as it complicates the understanding of genetic experimentations and restricts the selection of varieties adaptive to specific conditions (Farshadfar and Sutka, 2003). Normally, in plant breeding programs, the selection of genotypes for a specific environment is conducted by multi-environmental trials (METs) for the evaluation of genotypes based on their performance across environments (Li et al., 2020). Numerous research studies have been conducted using several statistical modeling approaches for checking the effect of GEI on yield and other agronomic traits (Grüneberg et al., 2005). These approaches mainly utilize a generalized linear model (GLM) to measure the variation caused by genotype, environment, and GEI for each variable by linear regression and joint analysis of variance (ANOVA) (Arif et al., 2021). GLMs lower the supposition of dependent variables (Olsson, 2002).

The additive main effect and multiplicative interaction (AMMI) model is mostly used in crop breeding programs for evaluating the genotypes for variety approval. First, the AMMI model uses ANOVA to divide variations into the main effect of genotype (G), main effect of environment (E), and effect caused by genotype-by-environment interaction (GEI). Second, it performs principal component analysis (PCA) by singular value decomposition for genotype and environment (Gauch Jr et al., 2008).

The most important method that visually helps to examine the relationship among genotypes, environments, and genotype-by-environment interaction and plays a significant role in the selection of the most stable and high-performing genotype for a specific environment in mega-environmental trials is the GGE biplot (Tiwari, 2019). GGE biplots play a major role in the selection of the most stable genotypes and discard those genotypes that are unstable across environments and/or have less yield (Li et al., 2020). In the past, many studies have been conducted to check the stability of soybean across environments. GGE biplots have been used to check the stability and adaptability of soybean genotypes that were cultivated in multiple environments and select the varieties that were highly stable and performed better across the environments (Mukuze et al., 2020; Carvalho et al., 2021). Other than soybean, GGE biplots were used in oat, sugarcane, rice, wheat, and maize for screening the stable genotypes in mega-environmental trials. Hence, it has been established that in agricultural research programs, the GGE biplot is the most effective method for the selection of suitable cultivars for specific environments in mega-environmental trials (Donoso-Ñanculao et al., 2016; Tena et al., 2019).

In general, genetic make-up (G), environment (E), and their interaction G × E influence the expression of any physiological and morphological trait. Due to their polygenic nature, yield and other quantitative traits are continuously controlled and affected by quantitative trait loci, genomic regions with associated genes, and environment (Said et al., 2022). As a result, genes that affect the yield and its components are highly sensitive to the environment and show QTL–environment interaction. This interaction between QTL and the environment can facilitate or constrain the responses toward artificial selection (Falconer, 1952). Therefore, breeding programs need to take these effects seriously and address them properly (El-Soda and Sarhan, 2021). Traditional QTL mapping and genome-wide association mapping are two methods that can be used for identifying the genes with an underlying natural variation that affects the genotypes.

In traditional breeding programs, the selection of genotypes is mostly carried out on the basis of phenotypic performance. Breeders mostly select the genotypes that perform well in a specific environment, costing time and resources (Baenziger, 2016). Association mapping (AM) is an alternative to conventional breeding and is considered an effective approach for dissecting the genomic location of genes or quantitative trait loci (Verdeprado et al., 2018). Based on the association between markers and traits, it performs rapid and fine mapping of the target locus (Mackay and Powell, 2007). In previous studies, association mapping conducted in soybean by using genome-wide SSR markers showed a successful marker–trait association (Ghione et al., 2021). The use of functional molecular markers, especially those derived from expressed sequence tags (ESTs), facilitates the association between phenotype and genotype by providing direct access to the population variation in genes of agronomically important traits (Mulato et al., 2010). In the past, linkage mapping was frequently used to check the effect of genotype, environment, and GEI (Ma et al., 2009). However, association mapping is rarely used for dissecting GEI (Lü et al., 2011). So, the aim of this study was 1) to check the effect of G × E interaction on the performance of soybean genotypes in multiple-environmental trials and select the genotypes that are most stable and have high adaptability across the environments, 2) identify the genotypes that give high yields in different environments, and 3) find the association between markers and traits for important agronomic traits.

## 2 Materials and methods

### 2.1 Plant material

A total of 96 soybean accessions acquired from USDA-ARS from different maturity groups (Supplementary Table S1) were selected. Of these accessions, eight were from Pakistan, including two locally adapted cultivars, Faisal soybean (G95) and Ajmeri (G96).

### 2.2 Experimental design

The field experiments were conducted at the Nuclear Institute of Agriculture (NIA) (25°′60′N 68°′60′E), Tando Jam, Sindh, Pakistan, and the National Institute for Biotechnology and Genetic Engineering (NIBGE), Faisalabad, Punjab (31°′42′N 73°′02′E), in the 2015–2016 growing season of soybean. The seeds were sown during August 2015 and 2016 at NIBGE and NIA and during August 2016 at NIBGE, with three replications of each accession. To analyze the data, each experiment was considered a separate environment. Experiments during February 2015/2016 at NIBGE were coded as E1 and E2, and those at NIA were E3 and E4, respectively. The experiment conducted during February 2016 at NIBGE was presented as E5.

**FIGURE 1:**
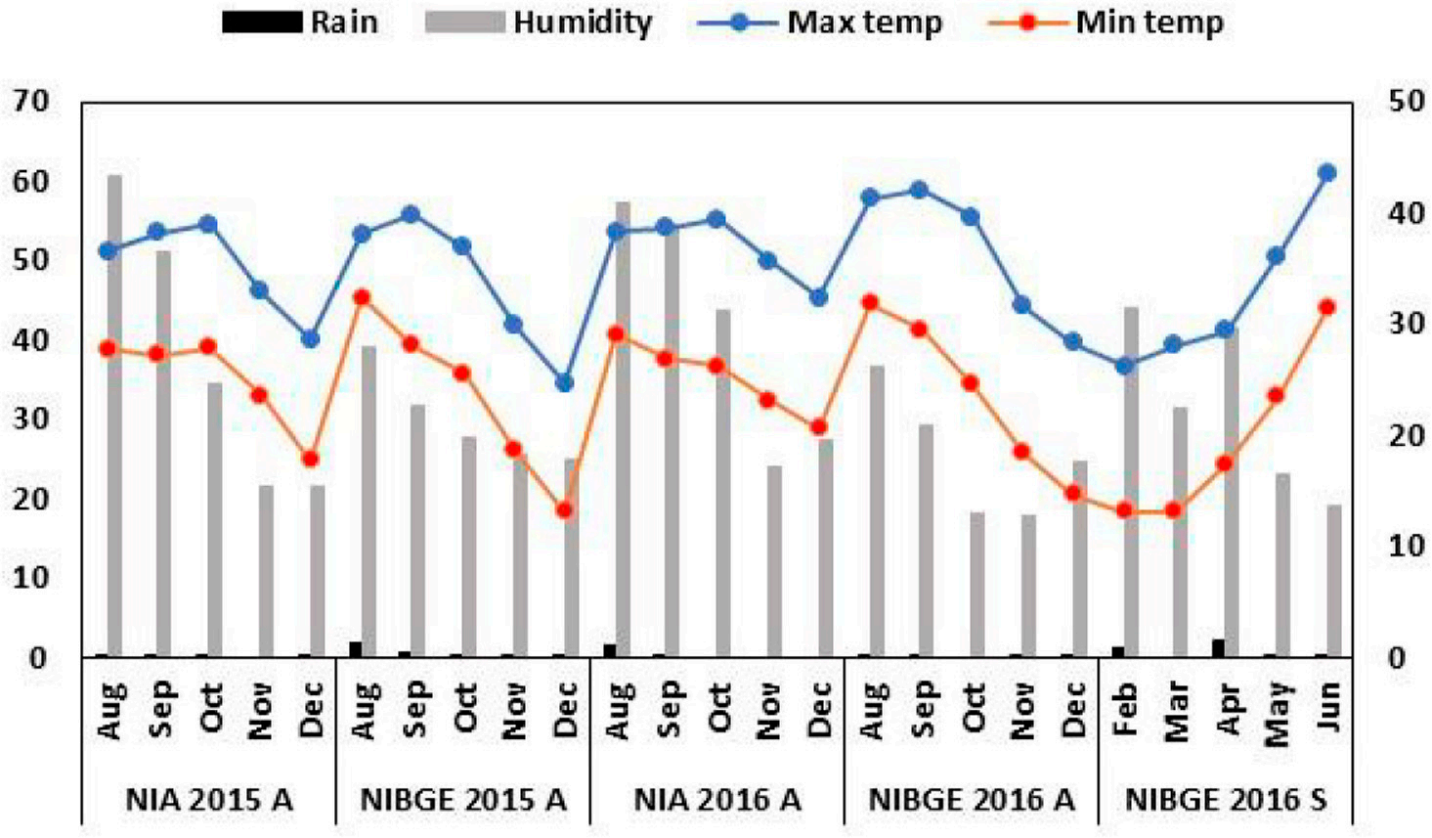
Weather footprint for the soybean genotypes’ growth period. Monthly rainfall (mm) (left x-axis) and relative humidity (%) (right x-axis).

The accessions were planted using a randomized complete block design. Seedbeds were prepared by one-time plowing with a cultivator, followed by planking and two-time plowing with a rotavator. To maintain a distance of three inches between plants, sowing was carried out with the help of a dibbler. Row to row distance of 30 cm and seed depth of 1-2 inches was maintained for proper emergence. Three rows of size 2.43 m were used for each soybean accession. Weather data for all the experimental locations were collected from https://www.worldweatheronline.com/ (Figure 1).

### 2.3 Phenotyping

Data were collected for plant height (cm), pods per plant (number), seeds per plant (number), seed weight per plant (gm), hundred-grain weight (gm), and total grain yield (gm). For phenotyping, the average data of three randomly selected plants were collected for each parameter except total yield. Plant height (PH) was measured at maturity from the surface of the soil to the tip of the plant. Pods per plant (PPP) were calculated for each randomly selected plant, and the average number of pod was recorded as pods per plant. Seed per plant (SDPP) was calculated as the average number of seeds present in three randomly selected plants of each accession. For seed weight per plant (SWPP), seeds of three randomly selected plants of each accession were weighed separately, and the average of three plants was recorded. For hundred-grain weight (HGW), 100 seeds were selected from the total seeds of each randomly selected plant, and the average of three plants was recorded as HGW. Total yield (TY) for each accession was measured after harvesting the whole plot.

### 2.4 Genotyping

For the association study, 100 genome-wide SSR markers were selected from the literature (Supplementary Table S2). For genotyping, DNA was extracted from young leaves using the method introduced by Doyle and Doyle (1987). PCR amplification was performed at an annealing temperature ranging from 42°C to 58°C, and the amplified product was run on 2.5% agarose gel. Scoring of bands was carried out on the basis of presence (0) and absence (1).

### 2.5 Phenotypic data analysis

#### 2.5.1 Correlation analysis

Correlation analysis between the six traits was performed in the web-based R software package “Performance Analytics” to find the significance of interrelationships between these traits based on Pearson’s correlation (Micheaux et al., 2013). By using the formula given by Wen et al. (2012), the correlation coefficient was calculated.

$$r=\frac{\overset{n}{\underset{i=1}{\sum }}\left(\;x\_i-├\;¯x\right)\left(y_{i}-¯y\right)\;}{\sqrt{\overset{n}{\underset{i=1}{\sum }}{\left(x_{i}-\bar{x}\right)}^{2}\;}\;\sqrt{{Ʃ}_{i=1}^{n}{\left(y_{i}-\bar{y}\right)}^{2}\;}},$$

where $\bar{x}$ and $\bar{y}$ denoted the mean value of *x*_*i*_ and *y*_*i*_ samples.

#### 2.5.2 Descriptive statistics

Descriptive statistics of six phenotypic traits was measured using the R-based package “metan,” which provides a simple and intuitive pipeline. The mean value of each variable was computed for all the combinations of genotypes and environments.

#### 2.5.3 Combined analysis of variance

The level of significance of the genotypes, environment, and their interaction in the multi-environment trial ANOVA was performed on six traits. In this model, a linear model along with the interaction effect was used, which is formulated as

$$y_{ijk}=\mu +\alpha_{i}+\tau_{j}+{\left(\alpha \tau \right)}_{ij}+\varepsilon_{ij.}$$

In this equation, *y*_*ijk*_ represents the response variable, which is observed in the *i*th genotype and *j*th environment; µ is used for the grand average; *α*_*i*_ represents the effect of the *i*th genotype; *τ*_*j*_ is the effect of the *j*th environment; (*ατ*)_*ij*_ represents the interaction effect of the *i*th genotype with the *j*th environment; and *ε*_*ij*_ is the residual standard error.

### 2.6 G × E data analysis

#### 2.6.1 AMMI and GLM models

In multi-environment experiments, GEI is commonly used to check the performance of genotype (G) across environments. The two statistical models used to evaluate the response of genotype in multi-environment are the AMMI model and GLM. In the AMMI method, ANOVA is used to access genotype G, environment E, and genotype × environment interaction to keep the genotype as fixed and the environment as a random effect, as described by Olivoto and Lúcio (2020). This method is further divided into interaction principal component analysis (IPCA) and AMMI main effect biplot analysis, where GE was plotted on the x-axis and IPCA values on the y-axis, while the second method is G and GEI biplot (Yan and Tinker, 2006).

$$y_{ij}=\mu +\alpha_{i\;}+\tau_{j}+\sum_{k=1}^{p}\;\lambda_{k}a_{ik}t_{jk}+\rho_{ij}+\varepsilon_{ij}.$$

Metan package of R is utilized to plot the data of GEI. On the other hand, MINITAB 14 software is used to perform GLM, which is a combination of ANOVA and generalized linear regression.

#### 2.6.2 GGE biplots

In METs, genotype main effect plus genotype-by-environment interaction (GGE) model were used for evaluation of appropriate genotype and environment. It can be written as

$$Y_{IJ\;}-\mu -\beta_{j\;}=\lambda_{1}\xi_{i1}\eta_{j1}+\lambda_{2}\xi_{i2}\eta_{j2}+\varepsilon_{ij},$$

where *Y*_*ij*_ stands for the average of the *i*th genotype in the *j*th environment; *µ* stands for the grand mean, and *β*_*j*_ stands for the main effect of the *j*th environment; *µ* + *β*_*j*_ is the mean variable of all the genotypes in the *j*th environment; *λ*_1_ and *λ*_2_ are singular values obtained from first two principal components (PC1 and PC2); *𝜉*_*i*1_ and *𝜉*_*i*2_ are the eigenvalues of PC1 and PC2 for *i*th genotype; *ɳ*_*j*1_ and *ɳ*_*j*2_ are eigenvectors of PC1 and PC2 for the *j*th environment, and *Ɛ*_ij_ is the residual for *i*th genotype and y, for *j*th environment.

### 2.7 Graphics

Trellis plots of phenotypic traits across the genotypes and environments were produced using Origin software.

### 2.8 Association mapping

Association analysis was conducted for individual environments for the evaluation of the association between markers and phenotypic traits and best linear unbiased prediction (BLUP) values using the GLM in TASSEL 3.0 software (Bradbury et al., 2007). Markers having a *p*-value of 1.03 × 10^–3^ are considered significantly associated with phenotypic traits. A linkage map was constructed based on the associated markers in Map chart software (Voorrips, 2002).

## 3 Results

### 3.1 Extent of phenotypic variations among environments

**FIGURE 2:**
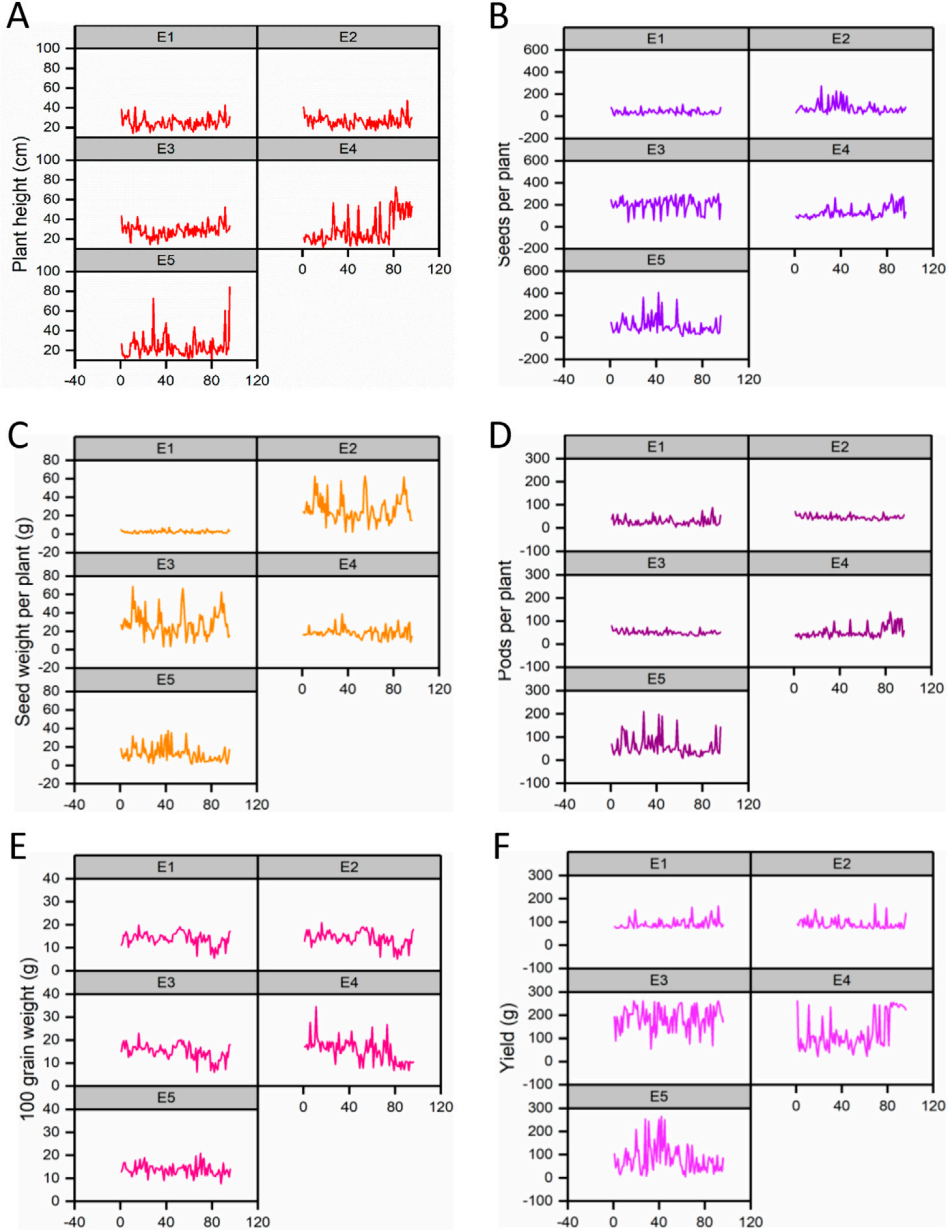
Variation in phenotypic data for traits is shown in trellis plots. **(A)** Plant height (PH). **(B)** Pods per plant (PPP). **(C)** Seeds per plant (SDPP). **(D)** Seed weight per plant (SWPP). **(E)** Hundred-grain weight (HGW). **(F)** Total yield (TY).

**FIGURE 3:**
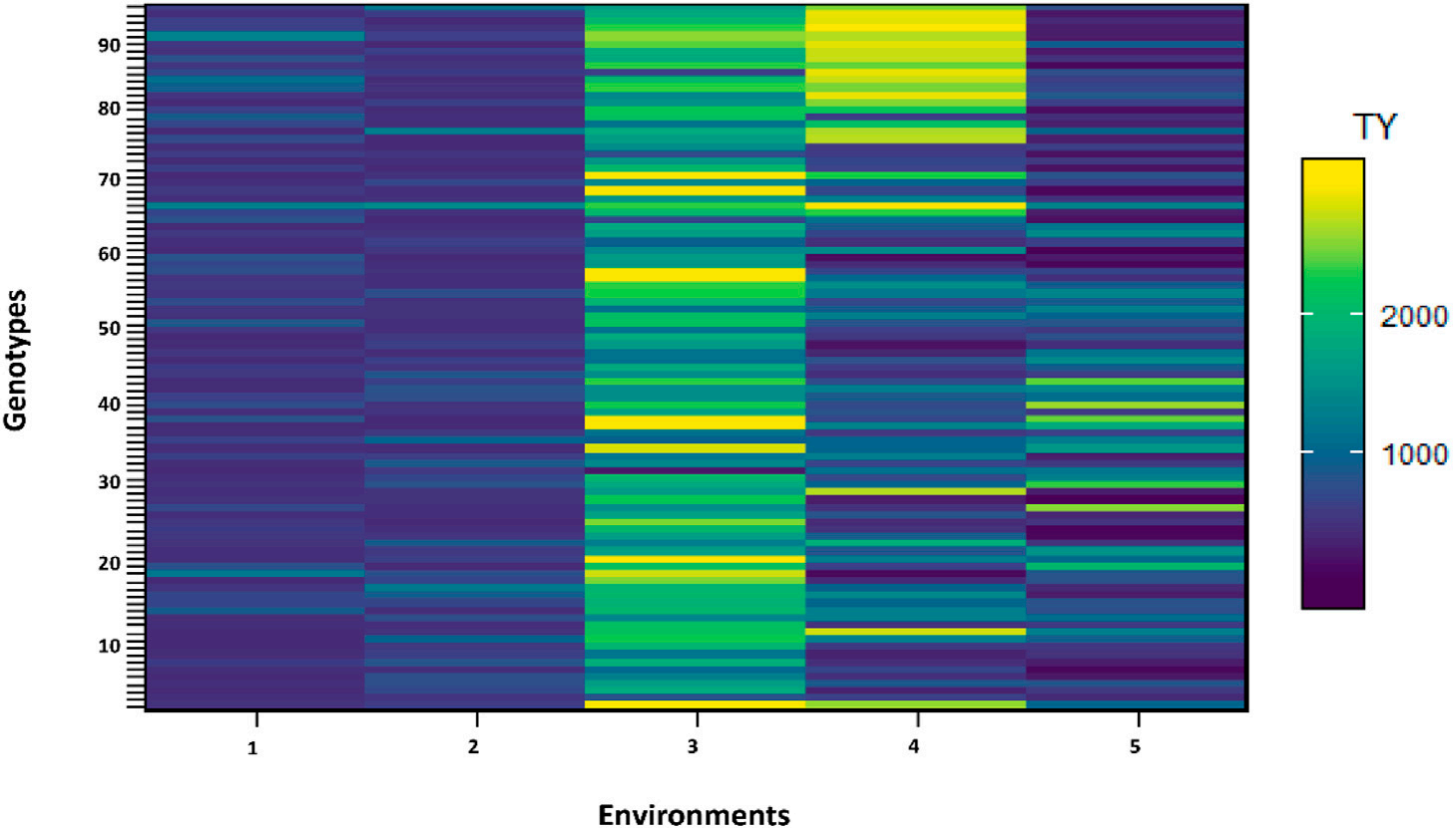
Shade plot across five environments. Number of genotypes (x-axis) and environments (y-axis).

The effect of each environment on morphological traits was measured and illustrated by trellis plots, which showed the significant effect of each on genotypes (Figure 2). Environments showed a clear effect on PPP, SDPP, SWPP, and TY; however, in the case of HGW and PH, genotypes were found to be the main source of variation. Based on the mean data of PPP, SDPP, and SWPP, E5 performed well in the spring season of Faisalabad. However, it was observed that TY was higher for most of the genotypes in E3 (Tando Jam) than other environments. Data recorded for TY presented higher variation for each genotype in all the environments (Figure 3).

### 3.2 Extent of genotypic variations among environments

**FIGURE 4:**
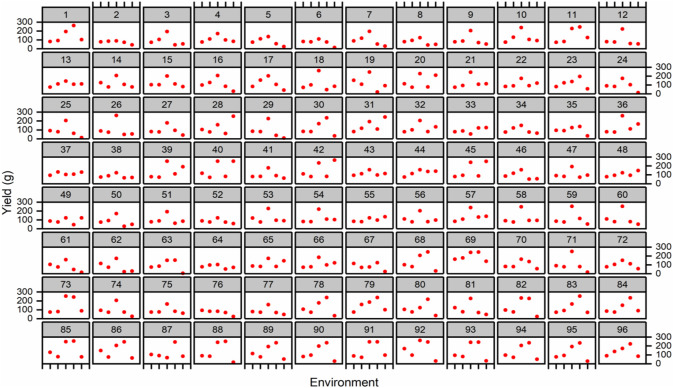
Trellis plots for average yield (g/plot) across five environments. Genotypes are coded as 1–96.

In multi-environment yield trials, it is common to represent a combination of cross and non-crossover types of genotype–environment interaction. Trellis plots demonstrated that average data of the total yield of individual genotypes were higher in E3 for the majority of genotypes when compared to other environments (Figure 4). Based on the trellis plots’ observation, G69 performed well in all the environments. However, G11, G69, G73, G82, G85, G88, G91, G92, and G3 were found to be stable in E3 and E4 and performed better during 2015/16 at Tando Jam.

### 3.3 Correlation analysis

**FIGURE 5:**
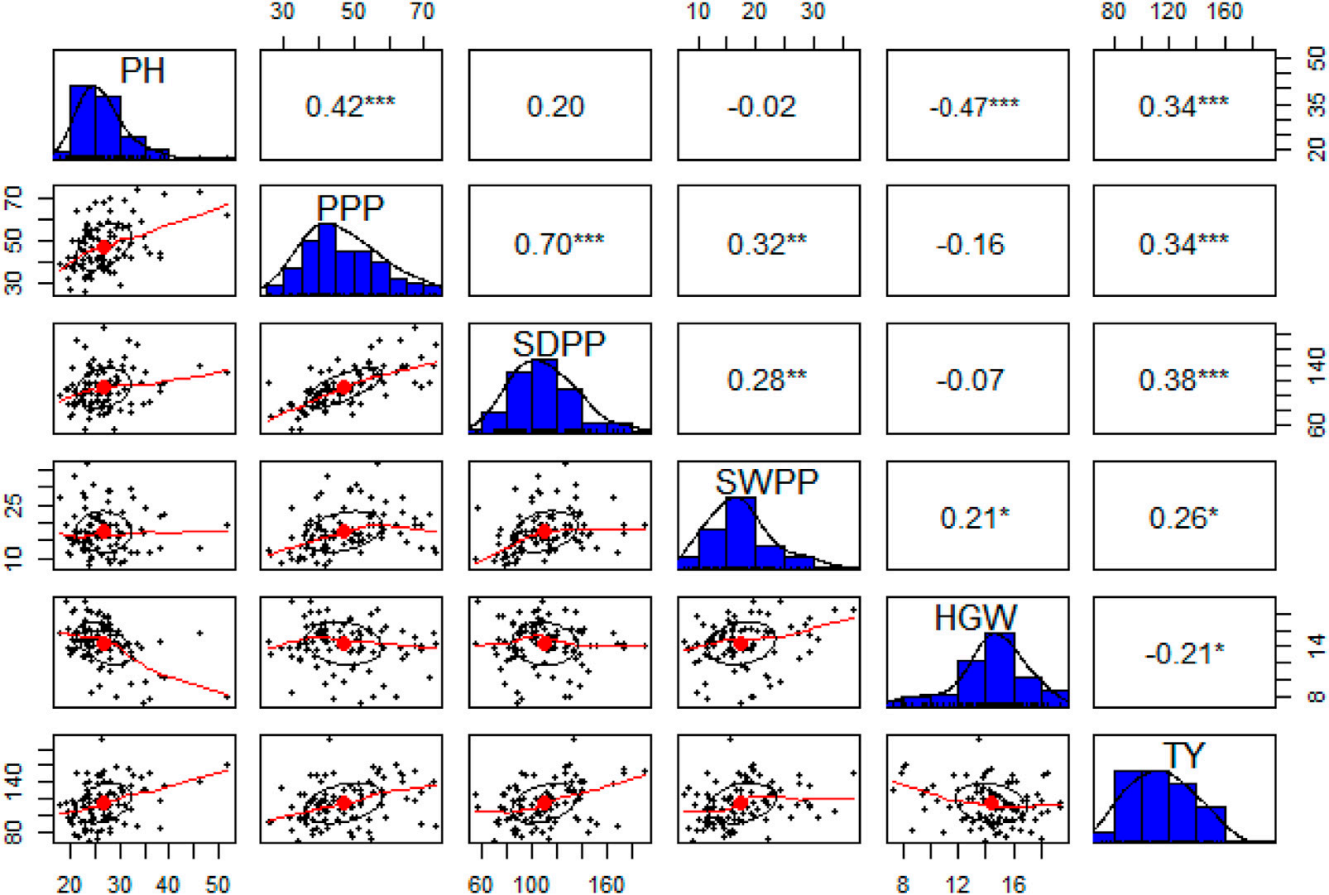
Correlation matrix (upper triangle), frequency distributions (blue bars), and bivariate scatter plots with a fitted line at lower triangle are shown for plant height (PH), pods per plant (PPP), seeds per plant (SDPP), seed weight per plant (SWPP), hundred-grain weight (HGW), and total yield (TY).

The pairwise correlation matrix of grand mean data of morphological traits showed a low level of positive correlation between PH, PPP, SDPP, SWPP, and TY. PPP and SDPP had a relatively high positive correlation as compared to other traits. HGW was negatively correlated with all the traits except SWPP, for which a low level of positive correlation was recorded. However, TY was positively correlated with all the traits at a low level except HGW (Figure 5).

### 3.4 G × E interaction

#### 3.4.1 Genotype main effects: AMMI biplots

**FIGURE 6:**
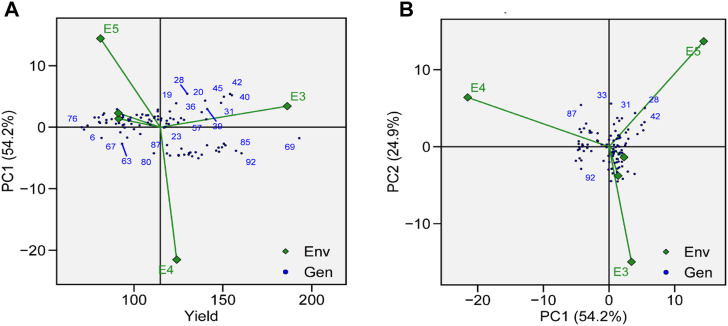
**(A)** AMMI main effects biplot for TY of genotypes across five environments. **(B)** The scatter plot of 96 soybean genotypes’ seed yield data across five environments explained 81.1% of the total variation. At the x-axis, PC1 explains (54.2%), and at the y-axis axis, PC2 explains (24.9%).

The identification of genotype adaptability to the nearby environment, i.e., broadly (near the origin) or specifically (far from the origin), can be carried out using AMMI main effect biplots (Figure 6A). Genotypes G19, G23, and G87 were less sensitive to environmental interaction in terms of seed yield as these were located near the origin. Similarly, the mean yield of G73, G11, G69, and G31 was found to be better than the grand mean yield of all genotypes. These genotypes were proposed to be high yielding and comparatively unresponsive to GEI. Performances of G2, G66, and G76 were less effective compared to the grand mean yield and were located near the origin along the y-axis but far from the origin on the x-axis, which means that these genotypes had low yield and were not affected by GEI. In a similar way, environments that had lower PCA scores and were found to be located near the central point along the y-axis had less contribution in GEI, such as E1 and E2, whereas E3, E4, and E5 showed strong interactive force. In terms of GEI, E4 had better contribution as it was plotted away from the center of origin along the x-axis. For TY, E4 was the most productive environment, followed by E3 and E5, and E1 and E2 were the least productive. Based on AMMI estimates in five environments, the ranks of 96 genotypes for the mean grain yield are presented in Supplementary Table S3.

#### 3.4.2 Genotype main effect: ANOVA (AMMI and GLM)

For better understanding, GLM was performed for our data along with the AMMI model, to compare the analytical competitiveness of GLM with special software-based AMMI analysis. Results obtained for ANOVA from both models were similar (Table 1). This suggests that genetic makeup of genotype has the least contribution in phenotypic variation of all traits compared to environment and GEI.

**TABLE 1:** Additive main effects and multiplicative interaction (AMMI) and generalized linear regression model (GLM) analysis of variance of the 96 soybean genotypes tested across five environments.

Source	Plant height (cm)				Pods per plant				Seeds per plant			
	AMMI	Var	GLM	Var	AMMI	Var	GLM	Var	AMMI	Var	GLM	Var
	SS	%	SS	%	SS	%	SS	%	SS	%	SS	%
Genotype (G)	43,388	31	14,465	31	172,982	18	57,099	18	958,167	11	321,594	11
Environment (E)	6,856	5	2,286	5	164,481	17	54,754	17	4,633,323	53	1,528,906	52
G x E	91,586	64	30,529	64	635,298	65	210,800	65	3,140,858	36	1,086,808	37
IPCA1	52,339	—	—	—	441,207	—	—	—	1,684,096	—	—	—
IPCA2	32,966	—	—	—	135,043	—	—	—	833,618	—	—	—
IPCA3	4,939	—	—	—	57,952	—	—	—	390,085	—	—	—
IPCA4	1,342	—	—	—	1,095	—	—	—	233,060	—	—	—
Residuals	18,362	—	—	—	167,387	—	—	—	283,773	—	—	—
Total	252,210	100	47,280	100	1,785,008	100	322,643	100	12,170,736	100	2,937,308	100

Source	Seed weight per plant (g)				Hundred-grain weight (g)				Total yield (g)			
	AMMI	Var	GLM	Var	AMMI	Var	GLM	Var	AMMI	Var	GLM	Var
	SS	%	SS	%	SS	%	SS	%	SS	%	SS	%
Genotype (G)	48,558	19	16,196	19	9,594	55	3,193	55	820,965	15	273,655	15
Environment (E)	125,820	49	41,925	49	636	4	205	4	2,138,951	38	712,984	38
G x E	82,002	32	27,367	32	7,216	41	2,455	41	2,640,105	47	880,035	47
IPCA1	62,152	—	—	—	5,220		—	—	1,431,114	—	—	—
IPCA2	15,156	—	—	—	1,659		—	—	658,172	—	—	—
IPCA3	3,889	—	—	—	279		—	—	439,416	—	—	—
IPCA4	805	—	—	—	57		—	—	111,403	—	—	—
Residuals	7,995	—	—	—	1,079		—	—	66,360	—	—	—
Total	348,233	100	85,488	100	25,945		5,852	100	8,322,069	100	1,866,674	100

The bolds provided are just to highlight the traits.

GEIs show how the performance of genotype is different in different environmental conditions. AMMI-based biplots were explained by the two interactive principal components. PCA 1 was on axis 1, while PCA 2 was on axis 2, and no GEI was explained by its origin. The scatter plot of TY data presented a negative correlation between E1, E2, E4, and E5, as shown by the obtuse angle between them (Figure 6B). Environments E1 and E2 were plotted near the origin and in the same cluster, elicited weak interactive forces, and had a similar influence on the genotypes, while E4 was located away from the origin and was subjected to strong interactive forces. Genotypes G69 and G72 were suggested to be under maximum GEI influence as they were located away from the center. G69 was located near E3 and was suggested to be specifically adapted for E3. G31, G41, and G42 were clustered together and had a similar yield across environments and were influenced by GEI in a similar way.

### 3.5 Selection of the best suitable genotype and environment

Various kinds of biplots can be drawn for better understanding of G × E analysis *via* GGE plots.

#### 3.5.1 Representativeness vs. discriminativeness

**FIGURE 7:**
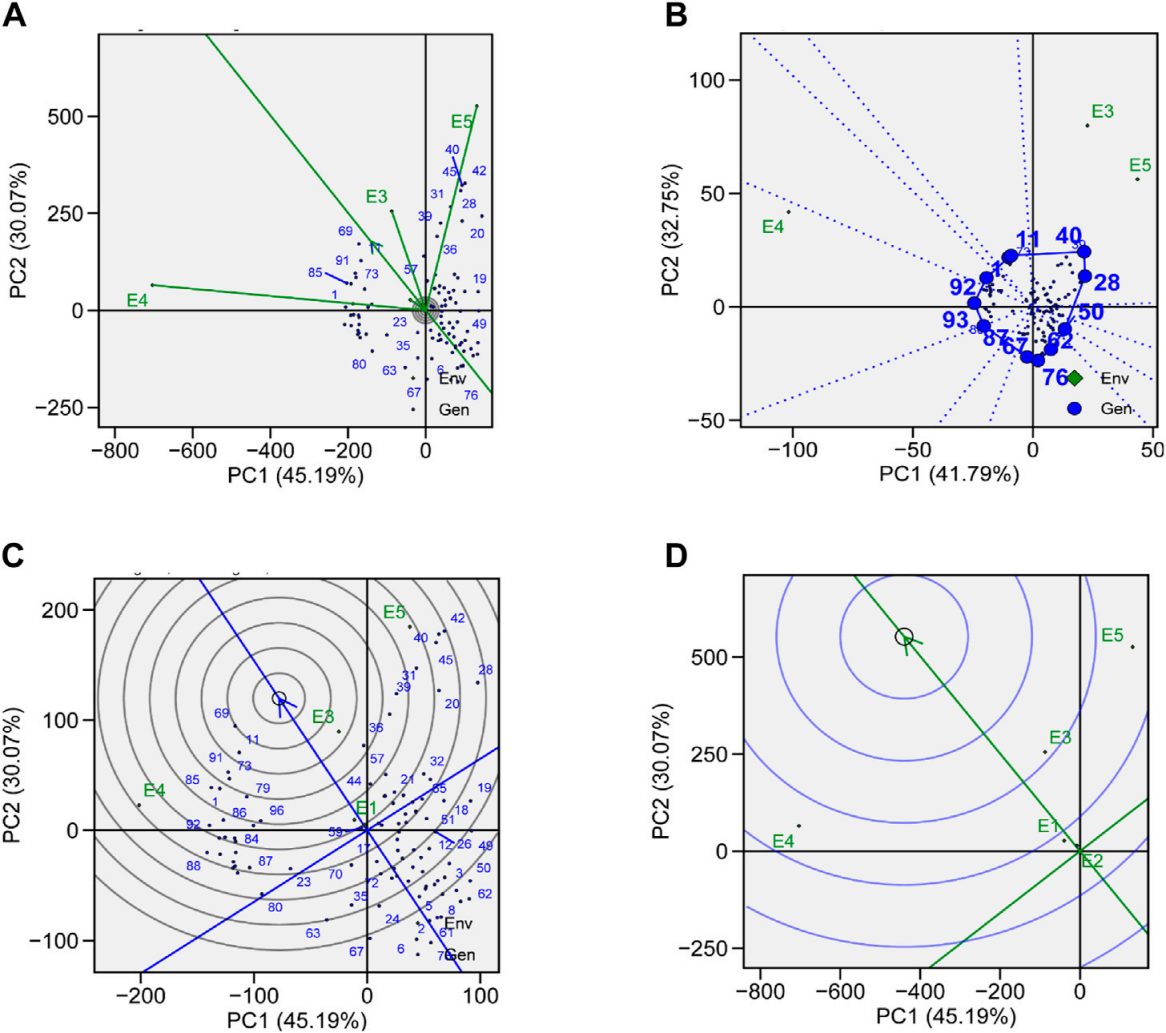
**(A)** Genotype plus genotype × environment interaction (GGE) biplot analysis for representation and discrimination of genotypes. **(B)** The which–won–where biplot for the yield of 96 soybean genotypes evaluated in five environments. **(C)** The best genotypes based on average performance and stability are displayed in a yield-focused comparative biplot. **(D)** Environment-focused comparison biplot explains the ideal environment for soybean yield among the locations used in evaluations.

To evaluate the genotypes with better and stable yield, representativeness and discriminative view of GGE biplots can be used on tested environments. The length of environmental vectors can be visualized, which is proportional to standard deviation in respective environments based on the concentric circle in the biplots and is a measure of the environmental ability to discriminate. Therefore, E3, E4, and E5 are the most discriminative, while E1 and E2 are less discriminative and provide very little information (Figure 7A). E1 and E2 are highly representative, based on the angle formed between the environmental vector and the average environment coordinate (AEC) axis. The smaller the angle between the environmental vector and AEC, the stronger will be the representativeness. Environments which are discriminating but non-representative are good for the selection of specifically adapted genotypes in mega-environments.

#### 3.5.2 Which–won–where

The which–won–where view of GGE biplot for TY helps in the identification of suitable genotypes for a specific environment in mega-environments. In our study, we observed three mega-environments: E3 and E5 formed mega-environment 1 (ME1), E1 and E2 formed mega-environment 2 (ME2), while E5 alone was mega-environment 3 (ME3) (Figure 7B). The polygon connects all the genotypes which are further from the origin of the biplot in such a way that all the genotypes are contained inside the polygon. Perpendicular lines generated from the center of origin help to compare the genotypes. Generally, the genotype that appears in the same sectors as the specific environment performs the best in that environment. The equality line that connects the adjacent genotypes on the polygon helps in visual comparison of the genotypes, e.g., the equality line that is formed between G11 and G40 shows that G40 was better in E3 and E5, while G11 performed better in other environments. So, these genotypes are expected to produce the maximum yield in that particular environment.

#### 3.5.3 Ranking genotypes relative to ideal genotype

A genotype that is highly stable across the environments and also has high mean performance is considered an ideal genotype. The performance of a genotype in a particular environment is ranked by the axis line that passes through center of origin. An ideal genotype is mostly plotted near the center of concentric circles to a point on the AEA (“absolutely stable”) in the positive direction. It also has a vector length that is equal to the longest vector of genotypes on the positive side of AEA (“highest mean performance”). In our case, G11 was considered more desirable than G69, even though G69 has a higher average yield (Figure 7C). G76 was considered to be the poorest of all the genotypes as it was the furthest from the center of the concentric circle and was consistently the poorest. Although the yield of G76 was very low, its performance was also stable.

As there was not a single genotype that produced the highest yield in all the environments, we selected top-20 high-yielding genotypes from each environment as a representative of high-yielding genotypes for that environment. If a genotype was one of the 20 high-producing genotypes in at least two environments, it was then selected. Consequently, 13 genotypes were identified and selected (Table 2).

**TABLE 2:** Top genotypes for high yields across five environments.

	E1	E2	E3	E4	E5
G92	*		*	*	
G69	*	*	*	*	*
G19	*		*		
G86	*			*	
G85	*		*	*	
G40	*		*		*
G20	*				*
G42	*				*
G31		*			*
G44		*			*
G36			*		*
G73			*	*	
G39			*	*	
Total	8	3	7	6	7

#### 3.5.4 Ranking environments

A ranking environment view of the GGE biplot is the most suitable method to check ideal environment for the selection of genotypes that perform better in a specific environment. The environment that is plotted near the concentric circle is more informative than those plotted far away from the center. So, in this case, E3 is suggested to be more ideal environment as it is plotted near the concentric circle and is very informative for the selection of genotypes with high yield (Figure 7D), while E1 and E2 are far away from the concentric circle and give very little information for selection of high-yielding genotypes.

### 3.6 Combined analysis of variance

Results obtained from combined ANOVA showed that the environment has the main influence on SDPP, SWPP, and HGW, whereas GEI has a high influence on TY, PH, and PPP, that is, 47%, 57%, and 56%, respectively (Table 3). The results obtained from AMMI-based ANOVA also showed that G × E has a major influence on TY, PH, and PPP, which showed that in soybean, genotypes performed differently across different environments, which may be because of differences in locations.

**TABLE 3:** Analysis of variance (ANOVA) combined for total yield (TY), plant height (PH), pods per plant (PPP), seeds per plant (SDPP), seed weight per plant (SWPP), and hundred-grain weight (HGW).

Traits	Source	Df	SS	Var %	MS	F value
Total yield (g)	Environment (E)	4	2,138,951.31	38	534,737.83	7,655.17
	Genotype (G)	95	820,965.26	14	8,641.74	123.71
	G × E	380	2,640,104.76	47	6,947.64	99.46
	Residuals	950	66,360.50	1	69.85	
	Total	1,429	5,666,381.83	100		
Plant height (cm)	Environment (E)	4	6,855.58	4	1713.90	88.67
	Genotype (G)	95	43,388.16	27	456.72	23.63
	G × E	380	91,586.43	57	241.02	12.47
	Residuals	950	18,361.64	12	19.33	
	Total	1,429	160,191.82	100		
Pods per plant	Environment (E)	4	164,481.43	14	41,120.36	233.38
	Genotype (G)	95	172,982.32	15	1820.87	10.33
	G × E	380	635,297.58	56	1,671.84	9.49
	Residuals	950	167,387.14	15	176.20	
	Total	1,429	1,140,148.46	100		
Seeds per plant	Environment (E)	4	4,633,323.10	51	1,158,330.78	3,877.80
	Genotype (G)	95	958,166.57	11	10,085.96	33.77
	G × E	380	3,140,858.43	35	8,265.42	27.67
	Residuals	950	283,772.90	3	298.71	
	Total	1,429	9,016,120.99	100		
Seed weight per plant (g)	Environment (E)	4	125,820.14	48	31,455.04	3,737.66
	Genotype (G)	95	48,558.27	18	511.14	60.74
	G × E	380	82,002.25	31	215.80	25.64
	Residuals	950	7,994.91	3	8.42	
	Total	1,429	264,375.57	100		
Hundred-grain weight (g)	Environment (E)	4	635.83	3	158.96	140.00
	Genotype (G)	95	9,594.31	52	100.99	88.95
	G × E	380	7,215.98	39	18.99	16.72
	Residuals	950	1,078.67	6	1.14	
	Total	1,429	18,524.80	100	1.14	

### 3.7 Analysis of variability, heritability, and genetic advance

From the results obtained from the genotypic coefficient of variance (GCV), phenotypic coefficient of variance (PCV), heritability, and genetic advance (GA) (Table 4), there is sufficient scope for selecting desired germplasm for each environment based on the agronomic traits. Both GCV% and PCV% calculated for PH, PPP, SDPP, SWPP, and HGW were higher in most of the environments, except for SDPP and HGW, where less distance was calculated between GCV and PCV in E1 (57.38/57.65), E3 (30.36/31.12), and E4 (39.46/39.70) for SDPP and E3 (22.15/22.58), E4 (31.91/32.15), and E5 (17.19/17.77) for HGW, respectively. In case of TY, distance calculated between PCV and GCV was very low in all environments. Heritability estimated in this study were PH (55%–92%), PPP (57%–97%), SDPP (82%–99%), SWPP (32%–98%), HGW (72.34–98), and TY (95%–99%). TY (99%) showed relatively higher heritability than other traits in all the environments. Maximum genetic advance (145) was calculated for TY in E4.

**TABLE 4:** Variability, heritability, and genetic advance estimate for six agronomic traits.

Traits	Environment	SEM	ECV (%)	GCV (%)	PCV (%)	h^2^%	GA
Plant height	E1	2.7024	18.72	20.7736	27.9639	55.19	7.9487
	E2	2.4832	16.1988	19.5046	25.3541	59.18	8.2068
	E3	1.9517	11.8329	22.1595	25.1209	77.81	11.5037
	E4	3.3932	19.789	49.5019	53.3108	86.22	28.1221
	E5	1.8496	13.4616	48.1595	50.0055	92.75	22.7386
Pods per plant	E1	1.6544	10.4287	58.3768	59.301	96.91	32.5277
	E2	3.5833	13.1744	19.332	23.3943	68.29	15.5033
	E3	2.9551	10.2789	18.4711	21.1385	76.35	16.5566
	E4	11.2558	37.1468	42.8019	56.6735	57.04	34.9485
	E5	11.944	34.8605	67.6765	76.1273	79.03	73.5491
Seeds per plant	E1	1.2216	5.6103	57.3823	57.6559	99.05	44.3704
	E2	8.1589	20.3741	62.3657	65.6093	90.36	84.7051
	E3	8.0271	6.8488	30.3627	31.1256	95.16	123.8603
	E4	3.3165	4.3303	39.4654	39.7023	98.81	107.2044
	E5	18.8243	31.733	68.8557	75.8162	82.48	132.3587
Seed weight per plant	E1	0.3142	21.2268	55.6528	59.5638	87.30	2.7458
	E2	2.1919	14.3328	49.8399	51.8599	92.36	26.1365
	E3	1.2055	7.5147	48.0508	48.6349	97.61	27.1735
	E4	1.824	8.851	17.1982	27.5685	32.493	0.7199
	E5	2.0839	29.2044	64.6533	70.9433	83.05	15.0015
Hundred-grain weight	E1	0.6169	7.6845	19.6252	21.0762	86.71	5.234
	E2	1.0575	13.0592	21.118	24.8297	72.34	5.1895
	E3	0.3755	4.3948	22.1574	22.5891	96.21	6.6256
	E4	0.3533	3.9428	31.912	32.1547	98.50	10.1245
	E5	0.3575	4.5005	17.1955	17.7747	93.59	4.7146
Total yield	E1	1.7858	3.3856	21.4582	21.7236	97.57	39.8917
	E2	1.5197	2.8868	22.8897	23.071	98.43	42.6546
	E3	2.1067	1.9595	26.9173	26.9885	99.47	102.985
	E4	9.3396	13.0413	58.3983	59.8367	95.25	145.6353
	E5	4.3883	9.3684	72.3745	72.9784	98.35	119.9615

### 3.8 Association mapping

**FIGURE 8:**
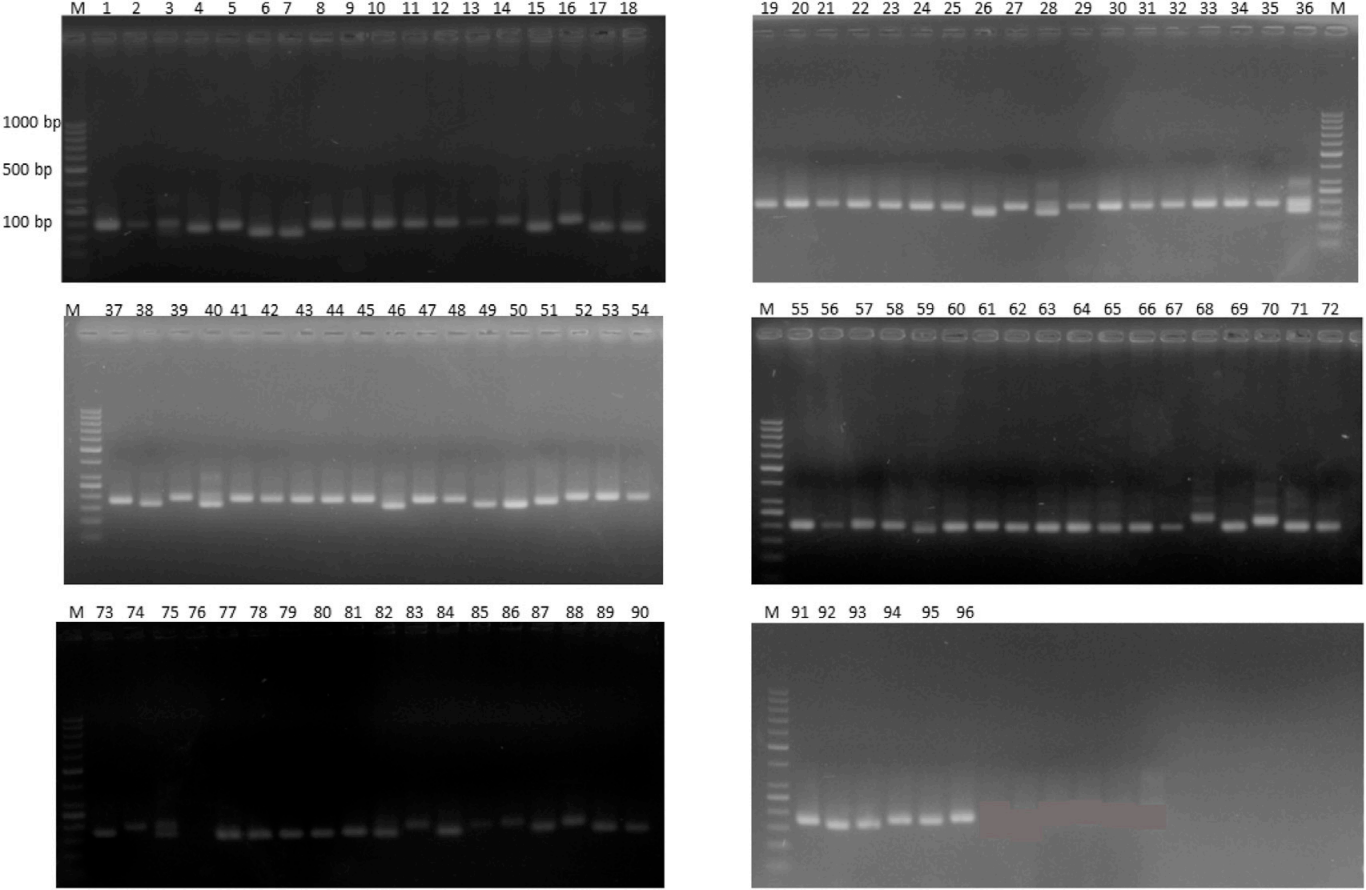
Representative gel image of 18 soybean genotypes with SSR marker Satt565 on 2.5% agarose gel. Lane M shows 1 kb plus DNA.

**FIGURE 9:**
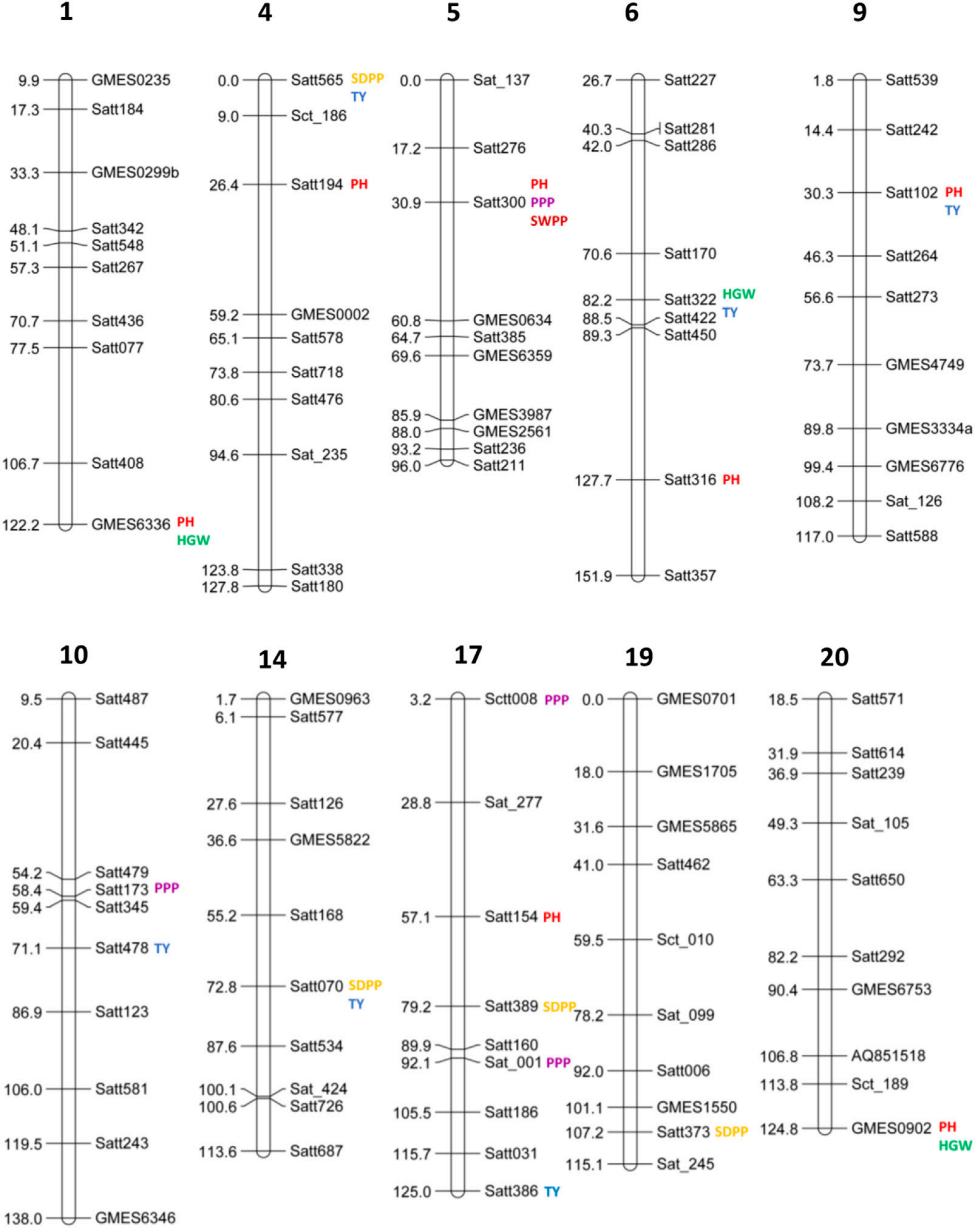
Genetic linkage map of soybean using simple sequence repeat (SSR) markers showing the marker positions and estimated map distance in cm on chromosomes 1, 4, 5, 6, 9, 10, 14, 17, 19, and 20. Markers associated with plant height (PH), pods per plant (PPP), seeds per plant (SDPP), seed weight per plant (SWPP), hundred-grain weight (HGW), and total yield (TY) are identified by colors. Markers that do not show any significant association with traits are not highlighted with any color.

Out of 100 markers, 96 were polymorphic, which produced a total of 262 alleles with an average of 2.79 alleles per locus (Figure 8). The average polymorphism information content of the molecular markers was 0.44, and 28 markers showed a PIC value ≥ 0.50. In five environments, a total of 26 marker–trait associations were found for six agronomic traits. The level of significance was set at *p* < 1.03 × 10^–3^ for identifying significant markers (Table 5). Most of the significant markers were found to be associated with a single trait in a single environment. SSR marker Satt316 was found to be associated with plant height at both locations during 2016 at NIBGE and 2015 at NIA with 12% of the total variation. Few markers like GMES0902, Satt565, GMES6336, Satt300, Satt322, Satt102, and Satt070 are associated with more than one trait, which may be due to positive correlations present among these traits. Two markers, Satt565 and Satt070, were associated with both seeds per plant and total yield. A total of nine markers were significantly associated with plant height, which explained 12%–31% of variation, while a single marker–trait association was observed for seed weight per plant with 18% of total variation. Six marker–trait associations were identified for the total yield at the tested environment with the total percentage of variation explained by each marker ranging from 12% to 19%. Most of the markers associated with the agronomic traits were located on chromosome 17. The genetic linkage map was constructed to depict the position of observed SSR and EST SSR markers (Figure 9).

**TABLE 5:** Marker–trait associations detected using GLM with six traits.

Trait	Marker	LG	Position (cM)	Environment	*p*-value	*R* ^2^
PH	Satt194	C1	26.35	V	2.48E-08	0.31
	GMES0902	I	124.8	IV	4.86E-04	0.12
	GMES6336	D1a	122.2	IV	5.26E-04	0.14
	Satt102	K	30.28	II	7.09E-04	0.15
	Satt300	A1	30.93	V	9.82E-04	0.18
	satt154	D2	57.07	V	1.18E-03	0.14
	Satt316	C2	127.66	**II**	2.36E-03	0.12
	Satt316	C2	127.66	**III**	2.80E-03	0.12
HGW	GMES0902	I	124.8	IV	3.04E-04	0.13
	GMES6336	D1a	122.2	IV	1.03E-03	0.14
	Satt322	C2	82.23	IV	1.39E-03	0.13
PPP	Satt173	O	58.4	I	4.10E-04	0.18
	Satt300	A1	30.93	V	1.04E-03	0.18
	Sat_001	D2	92.12	I	2.09E-03	0.15
	Sctt008	D2	3.2	V	2.99E-03	0.12
SWPP	Satt300	A1	30.93	V	1.03E-03	0.18
TY	Satt478	O	45	I	5.61E-04	0.19
	Satt102	K	30.28	V	1.10E-03	0.14
	Satt565	C1	16	V	1.48E-03	0.15
	Satt386	D2	125	I	1.93E-03	0.12
	Satt322	C2	82.23	V	2.91E-03	0.12
	Satt070	B2	72.8	I	2.98E-03	0.15
SDPP	Satt070	B2	72.8	II	7.95E-04	0.19
	Satt373	L	87	IV	1.67E-03	0.2
	Satt389	D2	79.23	II	2.27E-03	0.2
	Satt565	C1	0	II	2.68E-03	0.14

## 4 Discussion

The main objective of any breeding program is to develop genotypes that are resistant to biotic and abiotic stresses with high production. Environmental interaction has a significant impact on complex quantitative traits with several contributing factors such as yield. The existence of a substantial genotype main effect and G × E interactions revealed that genotypes respond differently in different environmental conditions. Studies on stability and GEI are crucial for effective breeding and adaptability in a wide range of environmental conditions (Liang et al., 2015). For the allocation of best resources in breeding or cultivar evaluation programs, the mega-environment concept is helpful (Gauch Jr and Zobel, 1997). A mega-environment is a collection of places that regularly use the same top cultivars (Yan and Rajcan, 2002). Because of the significant impact of genotype by mega-environment interaction, the evaluation of cultivars should be carried out separately for each mega-environment before the cultivar recommendation (Yan et al., 2011). Genotypes selected from the ideal test environment were mostly those with exceptional mean performance and greater adaptability (Yan et al., 2000). For identification of lines with high homeostasis in multilocation trials and coordinated variety testing programs, stability analysis models such as YSi statistics, AMMI, and GGE biplots were used. The main issue for plant breeders is to get the relevant knowledge concealed in multi-environment data and then to understand it for successful utilization. For mega-environment and cultivar evaluation, and assessment of varietal stability, GGE biplots have mostly been used (Rakshit et al., 2012; Zimmer et al., 2016). The GGE biplot was more beneficial when the mega-environment was used to evaluate a large set of genotypes, as the pattern of GEI could make the genotype evaluation more challenging (Krishnamurthy et al., 2017). In other words, environmental variation was inconsistent with the superiority of genotype, which restricted the selection of cultivars. The quality of selection can be improved by the selection of superior genotypes, with little stability variance produced through simultaneous selection for high mean and stability. In many crops, this technique has been effectively used, particularly for determining grain yield.

In this study, soybean genotypes were analyzed in five different environmental conditions, and the features that substantially correlated with stability were discussed. With the help of stability analysis models, stable genotypes with high mean yields were identified. These models suggested that the most stable genotype for TY was G69, followed by G92, G85, and G40 (Figure 6A). A genotype with a high mean yield and great stability would be an ideal genotype. A genotype located closer to the mean environment’s direction and having a projection of zero onto the perpendicular AEC coordinate is considered an “ideal” genotype. For mean yield and stability across environments, lines G69 and G92 displayed high mean rankings and were determined to be the best-performing ones (Figure 7C). Results obtained from GGE biplots suggested that G92 and G69 are most suited to E1, G69 to E2, G18 to E3, G1 to E4, and G42 to E5 (Figure 7B). These results are in line with the findings of Arif et al. (2021), who reported that genotypes DCD, BRC-457, and D-14005 were ideal genotypes for E5 as they have high mean yield and stability.

A significant variation was observed in the yield of genotypes, which may be due to the presence of diversity across environments. Similar results were reported by many researchers (Kumar et al., 2014; Bhartiya et al., 2017). Carvalho et al. (2021) also reported the epistatic effect on yield. Results obtained by GGE biplot analysis recommended that E3 was the most suitable environment for the selection of high-yielding genotypes and general adaptability (Figure 7D). E4 was the most discriminating and least representative environment for genotype evaluation and would be helpful in choosing genotypes that are specifically adapted (Mulugeta et al., 2013). The environment with the least discrimination but the most representation for the majority of attributes was E3 (Figure 7D). The large environmental difference suggested that there were genotypic variations in adaptation (Krisnawati and Adie, 2018). Regarding the stability of yield attributes, genotypes also varied greatly. Genotype G69 stood out in all evaluated environments (Table 2). However, when average yield was considered, G92, G85, and G40 performed better. Results obtained from this study are aligned with those from the work of AbdulHamid et al. (2017) and Nagarajan et al. (2017), who showed the importance of cultivating soybean genotypes with yield-contributing traits.

With the advancement of phenotyping and genotyping technologies, it has become easy to analyze the genomic regions related to quantitative traits in larger populations. Considering the relatively important interactions between the environments and genotypes, association mapping was performed for yield-contributing traits with SSR and EST-SSR markers for each environment separately. For six agronomic traits, 26 marker–trait associations were found in five environments (Table 4). No common markers among environments were discovered, which may be due to absence or very weak significant relationships between environments for TY. Additionally, many studies have demonstrated that variations in the size and structure of the population might affect the outcomes of association mapping (Liu and Cheng, 2020). Given that a smaller population offers fewer allelic types, genetic drift may be the cause of this variation. Another possible source could be the type and number of SSRs, as a preference for single-locus SSR in the present study may miscalculate the value after lowering the complexity of genotyping (Vigouroux et al., 2002). A typical population for an association study should consist of multiple unrelated and independent individuals from the same origin (Porth et al., 2013). Therefore, in order to eliminate false positive associations, we still need to confirm the association results by targeting allelic variations in coding regions *via* molecular biology approaches such as knockout studies (Abdurakhmonov and Abdukarimov, 2008) that offer a high-precision estimation of allelic variation.

## 5 Conclusion

From this study, we concluded that significant genetic variation was present between the genotypes for yield in different environments. In total, 13 environment-specific genotypes showing their maximum grain yield in each environment were identified. Genotype G69 was an ideal genotype with higher grain yield and broad adaptation to environments E1 and E2. In the case of environments, E3 was a more ideal environment as it was plotted near the concentric circle and was very informative for the selection of genotypes with high yield. Furthermore, association mapping revealed a total of 26 marker–trait associations for six agronomic traits in five environments, with the highest significance for plant height and the lowest significance for hundred-grain weights. As the G × E interaction has a significant effect on yield, it is necessary to further evaluate the ideal location for introducing suitable genotypes with stable high yield. Plant breeders must concentrate on improving features with high heritability.

## Data Availability

The original contributions presented in the study are included in the article/Supplementary Material; further inquiries can be directed to the corresponding authors.

## Abbreviations

AEC, average environment coordinate; AMMI, additive main effect and multiplicative interaction; ANOVA, analysis of variance; BLUP, best linear unbiased prediction; ECV, environment coefficient of variance; GCV, genotypic coefficient of variance; GEI, genotype–environment interaction; GLM, general linear model; HGW, hundred-grain weight; IPCA, interaction principle component analysis; LG, linkage group; PCV, phenotypic coefficient of variance; PH, plant height; PPP, pods per plant; R2, coefficient of determination; SDPP, seeds per plant; SEM, standard error of the mean; SWPP, seed weight per plant; TY, total yield.

## References

[B1] Abdulhamid M. Qabil N. El-Saadony F. (2017). Genetic variability, correlation and path analyses for yield and yield components of some bread wheat genotypes. J. Plant. Prod. 8, 845–852. 10.21608/JPP.2017.40877

[B2] Abdurakhmonov I. Y. Abdukarimov A. (2008). Application of association mapping to understanding the genetic diversity of plant germplasm resources. Inte J. Plant Genomics 2008, 574927. 10.1155/2008/574927 PMC2423417 18551188

[B3] Arif A. Parveen N. Waheed M. Q. Atif R. M. Waqar I. Shah T. M. (2021). A comparative study for assessing the drought-tolerance of chickpea under varying natural growth environments. Front. Plant Sci. 11, 607869. 10.3389/fpls.2020.607869 33679816 PMC7928316

[B4] Baenziger P. (2016). Wheat breeding and genetics. Ref. Modul. Food Sci**.** 1–10. 10.1016/B978-0-08-100596-5.03001-8

[B5] Bhartiya A. Aditya J. Kumari V. Kishore N. Purwar J. Agrawal A. (2017). GGE biplot & ammi analysis of yield stability in multi-environment trial of soybean [*Glycine max* (L.) Merrill] genotypes under rainfed condition of north Western Himalayan hills. J. Anim. Plant Sci. 27, 227–238.

[B6] Bradbury P. J. Zhang Z. Kroon D. E. Casstevens T. M. Ramdoss Y. Buckler E. S. (2007). Tassel: Software for association mapping of complex traits in diverse samples. Bioinformatics 23, 2633–2635. 10.1093/bioinformatics/btm308 17586829

[B7] Carvalho M. P. Nunes J. a. R. Carmo E. L. D. Simon G. A. Moraes R. N. O. (2021). Adaptability and stability of conventional soybean by GGE biplot analysis. Pesqui. Agropecu. Trop. 51. 10.1590/1983-40632021v5167995

[B8] Debebe A. Singh H. Tefera H. (2014). Interrelationship and path coefficient analysis of yield components in F4 progenies of tef (Eragrostis tef). Pak. J. Biol. Sci. 17, 92–97. 10.3923/pjbs.2014.92.97 24783784

[B9] Donoso-Ñanculao G. Paredes M. Becerra V. Arrepol C. Balzarini M. J. C. J. O. A. R. (2016). GGE biplot analysis of multi-environment yield trials of rice produced in a temperate climate. Chil. J. Agric. Res. 76, 152–157. 10.4067/S0718-58392016000200003

[B10] Doyle J. J. Doyle J. L. (1987). A rapid DNA isolation procedure for small quantities of fresh leaf tissue. Phytochem. Bull. 19, 11–15.

[B11] Eltaher S. Baenziger P. S. Belamkar V. Emara H. A. Nower A. A. Salem K. F. (2021). GWAS revealed effect of genotype× environment interactions for grain yield of Nebraska winter wheat. BMC Genomics 22, 2–14. 10.1186/s12864-020-07308-0 33388036 PMC7778801

[B12] El‐Soda M. Sarhan M. S. (2021). From gene mapping to gene editing, A guide from the arabidopsis research. Annu. Plant Rev. online 4, 733–766. 10.1002/9781119312994.apr0765

[B13] Falconer D. S. (1952). The problem of environment and selection. Am. Nat. 86, 293–298. 10.1086/281736

[B14] Farshadfar E. Sutka J. (2003). Locating QTLs controlling adaptation in wheat using AMMI model. Cereal Res. Commun. 31, 249–256. 10.1007/bf03543351

[B15] Gauch H. G. Jr Piepho H. P. Annicchiarico P. (2008). Statistical analysis of yield trials by AMMI and GGE: Further considerations. Crop Sci. 48, 866–889. 10.2135/cropsci2007.09.0513

[B16] Gauch H. G. Jr Zobel R. W. (1997). Identifying mega‐environments and targeting genotypes. Crop Sci. 37, 311–326. 10.2135/cropsci1997.0011183X003700020002x

[B17] Ghione C. E. Lombardo L. A. Vicentin I. G. Heinz R. A. (2021). Association mapping to identify molecular markers associated with resistance genes to stink bugs in soybean. Euphytica 217, 46–12. 10.1007/s10681-021-02768-1

[B18] Grüneberg W. J. Manrique K. Zhang D. Hermann M. (2005). Genotype× environment interactions for a diverse set of sweetpotato clones evaluated across varying ecogeographic conditions in Peru. Crop Sci. 45, 2160–2171. 10.2135/cropsci2003.0533

[B19] Islam M. Sarker M. Sharma N. Rahman M. Collard B. Gregorio G. (2016). Assessment of adaptability of recently released salt tolerant rice varieties in coastal regions of South Bangladesh. Field Crops Res. 190, 34–43. 10.1016/j.fcr.2015.09.012

[B20] Krishnamurthy S. Sharma P. Sharma D. Ravikiran K. Singh Y. Mishra V. (2017). Identification of mega-environments and rice genotypes for general and specific adaptation to saline and alkaline stresses in India. Sci. Rep. 7, 7968. 10.1038/s41598-017-08532-7 28801586 PMC5554213

[B21] Krisnawati A. Adie M. M. (2018). Yield stability of soybean promising lines across environments. IOP Conf. Ser. Earth Environ. Sci. 102, 012044. 10.1088/1755-1315/102/1/012044

[B22] Kumar A. Kumar S. Kapoor C. Bhagawati R. Pandey A. Pattanayak A. (2014). GGE biplot analysis of genotype× environment interaction in soybean grown in NEH regions of India. Environ. Ecol. 32, 1047–1050.

[B23] Li M. Liu Y. Wang C. Yang X. Li D. Zhang X. (2020). Identification of traits contributing to high and stable yields in different soybean varieties across three Chinese latitudes. Front. Plant. Sci. 10, 1642. 10.3389/fpls.2019.01642 32038668 PMC6985368

[B24] Liang S. Ren G. Liu J. Zhao X. Zhou M. Mcneil D. (2015). Genotype-by-environment interaction is important for grain yield in irrigated lowland rice. Field Crops Res. 180, 90–99. 10.1016/j.fcr.2015.05.014

[B25] Liu N. Cheng F. (2020). Association mapping for yield traits in Paeonia rockii based on SSR markers within transcription factors of comparative transcriptome. BMC Plant Biol. 20, 245. 10.1186/s12870-020-02449-6 32487017 PMC7265254

[B26] Lü H. Y. Liu X. F. Wei S. P. Zhang Y. M. (2011). Epistatic association mapping in homozygous crop cultivars. PLoS One 6, e17773. 10.1371/journal.pone.0017773 21423630 PMC3058038

[B27] Ma L. Yang C. Zeng D. Cai J. Li X. Ji Z. (2009). Mapping QTLs for heading synchrony in a doubled haploid population of rice in two environments. J. Genet. Genomics 36, 297–304. 10.1016/S1673-8527(08)60118-6 19447378

[B28] Mackay I. Powell W. J. T. I. P. S. (2007). Methods for linkage disequilibrium mapping in crops. Trends Plant Sci. 12, 57–63. 10.1016/j.tplants.2006.12.001 17224302

[B29] Mukuze C. Tukamuhabwa P. Maphosa M. Dari S. Obua T. Kongai H. (2020). Evaluation of the performance of advanced generation soybean [*Glycine max* (L.) Merr.] genotypes using GGE biplot. J. Plant Breed. Crop Sci. 12, 246–257. 10.5897/JPBCS2020.0905

[B30] Mulato B. M. Möller M. Zucchi M. I. Quecini V. Pinheiro J. B. (2010). Genetic diversity in soybean germplasm identified by SSR and EST-SSR markers. Pesq. Agropec. Bras. 45, 276–283. 10.1590/S0100-204X2010000300007

[B31] Mulugeta A. Sisay K. Seltene A. Zelalem F. (2013). GGE biplots to analyze soybean multi-environment yield trial data in north Western Ethiopia. J. Plant Breed. Crop Sci. 5, 245–254. 10.5897/JPBCS13.0403

[B32] Nagarajan D. Kalaimagal T. Murugan E. (2017). Combining ability analysis for yield component and biochemical traits in soybean [*Glycine max* (L.) Merrill]. Int. J. Curr. Microbiol. Appl. Sci. 6, 2894–2901. 10.20546/ijcmas.2017.611.341

[B33] Olivoto T. Lúcio A. D. C. (2020). metan: An R package for multi‐environment trial analysis. Methods Ecol. Evol. 11, 783–789. 10.1111/2041-210X.13384

[B34] Olsson U. (2002). Generalized linear models. An applied approach. Lund: Studentlitteratur.

[B35] Porth I. Klapšte J. Skyba O. Hannemann J. Mckown A. D. Guy R. D. (2013). Genome‐wide association mapping for wood characteristics in P opulus identifies an array of candidate single nucleotide polymorphisms. New Phytol. 200, 710–726. 10.1111/nph.12422 23889164

[B36] Rakshit S. Ganapathy K. Gomashe S. Rathore A. Ghorade R. Kumar M. (2012). GGE biplot analysis to evaluate genotype, environment and their interactions in sorghum multi-location data. Euphytica 185, 465–479. 10.1007/s10681-012-0648-6

[B37] Said A. A. Macqueen A. H. Shawky H. Reynolds M. Juenger T. E. El-Soda M. (2022). Genome-wide association mapping of genotype-environment interactions affecting yield-related traits of spring wheat grown in three watering regimes. Environ. Exp. Bot. 194, 104740. 10.1016/j.envexpbot.2021.104740

[B38] Shrestha S. Asch F. Dusserre J. Ramanantsoanirina A. Brueck H. (2012). Climate effects on yield components as affected by genotypic responses to variable environmental conditions in upland rice systems at different altitudes. Field Crops Res. 134, 216–228. 10.1016/j.fcr.2012.06.011

[B39] Tariku S. Lakew T. Bitew M. Asfaw M. (2013). Genotype by environment interaction and grain yield stability analysis of rice (*Oryza sativa* L.) genotypes evaluated in north Western Ethiopia. Ethiopia. Net. J. Agri. Sci. 1, 10–16.

[B40] Tena E. Goshu F. Mohamad H. Tesfa M. Tesfaye D. Seife A. (2019). Genotype× environment interaction by AMMI and GGE-biplot analysis for sugar yield in three crop cycles of sugarcane (*Saccharum officinirum* L.) clones in Ethiopia. Cogent Food Agric. 5, 1651925. 10.1080/23311932.2019.1651925

[B41] Tiwari J. K. (2019). GGE biplot and AMMI model to evaluate spine gourd (Momordica dioica Roxb.) for genotype× environment interaction and seasonal adaptation. Electron. J. Plant Breed. 10, 264–271. 10.5958/0975-928x.2019.00031.0

[B42] Verdeprado H. Kretzschmar T. Begum H. Raghavan C. Joyce P. Lakshmanan P. (2018). Association mapping in rice: Basic concepts and perspectives for molecular breeding. Plant Prod. Sci. 21, 159–176. 10.1080/1343943X.2018.1483205

[B43] Vigouroux Y. Jaqueth J. S. Matsuoka Y. Smith O. S. Beavis W. D. Smith J. S. C. (2002). Rate and pattern of mutation at microsatellite loci in maize. Mol. Biol. Evol. 19, 1251–1260. 10.1093/oxfordjournals.molbev.a004186 12140237

[B44] Voorrips R. (2002). MapChart: Software for the graphical presentation of linkage maps and QTLs. J. Hered. 93, 77–78. 10.1093/jhered/93.1.77 12011185

[B45] Wen Z. P. Liu T. Zhang F. Liu N. Kang J. F. (2012). Comprehensive evaluation model for normal water level scheme based on GIS and grey correlation analysis. J. Zhejiang Univ. Sci. 39.

[B46] Yan W. Hunt L. A. Sheng Q. Szlavnics Z. (2000). Cultivar evaluation and mega‐environment investigation based on the GGE biplot. Crop Sci. 40, 597–605. 10.2135/cropsci2000.403597x

[B47] Yan W. Pageau D. Frégeau‐Reid J. Durand J. (2011). Assessing the representativeness and repeatability of test locations for genotype evaluation. Crop Sci. 51, 1603–1610. 10.2135/cropsci2011.01.0016

[B48] Yan W. Rajcan I. (2002). Biplot analysis of test sites and trait relations of soybean in Ontario. Crop Sci. 42, 11–20. 10.2135/cropsci2002.1100 11756248

[B49] Yan W. Tinker N. A. (2006). Biplot analysis of multi-environment trial data: Principles and applications. Can. J. Plant. Sci. 86, 623–645. 10.4141/P05-169

[B50] Yongchun L. Deyue Y. Ran X. (2008). Effects of natural selection of several quantitative traits of soybean RIL populations derived from the combinations of peking? 7605 and RN-9? 7605 under two ecological sites. Sci. Agric. Sin. 41.

[B51] Zimmer S. Messmer M. Haase T. Piepho H. P. Mindermann A. Schulz H. (2016). Effects of soybean variety and Bradyrhizobium strains on yield, protein content and biological nitrogen fixation under cool growing conditions in Germany. Eur. J. Agron. 72, 38–46. 10.1016/j.eja.2015.09.008

