# Supplementary material for: Analysis of genotype × environment interactions for agronomic traits of soybean (Glycine max [L.] Merr.) using association mapping
Source: Front Genet. 2023 Jan 5;13:1090994. doi: 10.3389/fgene.2022.1090994 (PMC9851276; doi:10.3389/fgene.2022.1090994)
Supplement: Supplementary file 1 [file Table1.DOCX]

Supplementary Material

# Supplementary Figures and Tables

## Supplementary Tables

**Supplementary Table S1.** Detail of Soybean Genotypes along with origin and maturity group.

| Genotype | PI number | Origin | Maturity Group | Genotype | PI number | Origin | Maturity Group |
| --- | --- | --- | --- | --- | --- | --- | --- |
| G1 | PI633609 | USA, Arkansan | V | G49 | PI644047 | USA, Georgia | IV |
| G2 | PI615694 | USA, South Carolina | VII | G50 | PI644053 | USA, Georgia | IV |
| G3 | PI661157 | USA, North Carolina | VII | G51 | PI644054 | USA, Georgia | III |
| G4 | PI641156 | USA, North Carolina | VII | G52 | PI644056 | USA, Georgia | III |
| G5 | PI634827 | USA, Nebraska | II | G53 | PI644057 | USA, Georgia | IV |
| G6 | PI614832 | USA, Nebraska | III | G54 | PI644058 | USA, Georgia | VII |
| G7 | PI612157 | USA, Georgia | V | G55 | PI644059 | USA, Georgia | VII |
| G8 | PI617041 | USA, South Carolina | VII | G56 | PI657825 | USA, Virginia | VII |
| G9 | PI665996 | USA, Illinois | IV | G57 | PI657826 | USA, Virginia | VII |
| G10 | PI628836 | Brazil | VII | G58 | PI657829 | USA, Virginia | VIII |
| G11 | PI628837 | Brazil | VI | G59 | PI659348 | USA, Mississippi | VIII |
| G12 | PI628838 | Brazil | V | G60 | PI542393 | USA | I |
| G13 | PI628875 | Brazil | VII | G61 | PI555464 | USA | VIII |
| G14 | PI628917 | Brazil | VI | G62 | PI054591 | China | VII |
| G15 | PI628918 | Brazil | VI | G63 | PI079693 | China | VII |
| G16 | PI595645 | USA, Georgia | VII | G64 | PI088788 | China | VII |
| G17 | PI628875 | Brazil | VII | G65 | PI090763 | China | VII |
| G18 | PI548660 | China | VII | G66 | PI091138 | China | VII |
| G19 | PI542712 | USA, Georgia | VI | G67 | PI232991 | China | V |
| G20 | PI548533 | USA, Illinois | IV | G68 | PI250844 | Iran | V |
| G21 | PI548445 | China | VII | G69 | PI371611 | Pakistan | I |
| G22 | PI548664 | USA, Florida | VIII | G70 | PI404153 | USA, Georgia | VIII |
| G23 | PI536009 | USA, Georgia | VII | G71 | PI404198A | China | III |
| G24 | PI553045 | USA, Georgia | VIII | G72 | PI437126A | USA, Georgia | II |
| G25 | PI535807 | USA, Texas | VIII | G73 | PI437127B | USA, Georgia | IV |
| G26 | PI614153 | USA, Ohio | III | G74 | PI437654 | China | III |
| G27 | PI508083 | USA, Minnesota | O | G75 | PI438183 | China | IV |
| G28 | PI553039 | USA, Arkansas | VI | G76 | PI438498 | USA | II |
| G29 | PI553041 | USA, Georgia | VII | G77 | PI475822A | China | I |
| G30 | PI548655 | USA, Mississippi | V | G78 | PI567209B | USA, Georgia | II |
| G31 | PI548657 | USA, Mississippi | V | G79 | PI166028 | India | IV |
| G32 | PI548389 | USA, North Carolina | V | G80 | PI171451 | Japan | IV |
| G33 | PI548482 | USA, Ohio | II | G81 | PI212604 | Afghanistan | III |
| G34 | PI548485 | USA, Mississippi | IV | G82 | PI212605 | Afghanistan | III |
| G35 | PI508084 | USA, Alabama | III | G83 | PI219698 | Pakistan | I |
| G36 | PI522236 | USA, Illinois | II | G84 | PI222397 | Pakistan | I |
| G37 | PI548493 | USA, Georgia | VII | G85 | PI269518C | Pakistan | - |
| G38 | PI548631 | USA, Georgia | VI | G86 | PI346309 | India | OO |
| G39 | PI518671 | USA, Illinois | I | G87 | PI371607 | Pakistan | IV |
| G40 | PI553042 | USA, Illinois | III | G88 | PI437126C | USA, Georgia | IV |
| G41 | PI553038 | USA, Ohio | VII | G89 | PI462312 | India | - |
| G42 | PI548271 | USA, North Carolina | IV | G90 | PI486328 | India | - |
| G43 | PI518771 | USA, Mississippi | VII | G91 | PI567203 | USA, Georgia | - |
| G44 | PI561701 | USA, Georgia | III | G92 | PI323278 | Pakistan | VII |
| G45 | PI591825 | USA, Georgia | VII | G93 | PNR791 | Australia |  |
| G46 | PI643914 | USA, Georgia | VIII | G94 | A6786 | Australia |  |
| G47 | PI644045 | USA, Georgia | IV | G95 |  | Pakistan |  |
| G48 | PI644046 | USA, Georgia | VI | G96 |  | Pakistan |  |

**Supplementary Table S2.** List f Markers and their sequence used in this study

| **Markers** | **Forward Primer** | **Reverse Primer** |
| --- | --- | --- |
| Satt636 | GTCATGACTCATGAGTCACGTAAT | CCCAAGACCCCCATTTTTATGTCT |
| Satt706 | GCGTTTTTTTCCCAACAATTAATTAGTATAT | GCGAAATGGTTATTATTGTTAAAAAATAATG |
| BE475343 | GCGTCTCCCTGTCTCTC | GCGAGCTTAAAACAATCATC |
| Satt316 | GTGAGAAACTAGCCAAGAATAGA | CAATTGTTTCCAAATGACACT |
| Sat-137 | GATGACGCCGCAGGTTTCTCC | GGTGCGGTTCCACAGTTTTTT |
| Satt385 | AATCGAGGATTCACTTGAT | CATTGGGCCACACAACAAC |
| Sat_334 | GCGTAACGTAGCAAATTGACTATAAGA | GCGTGTGCAAAGACAATTTCAATGA |
| Satt564 | GCGCTTCCACCACAATAACA | GCGGCAGAGGACTGACAGCTA |
| Satt045 | TGGTTTCTACTTTCTATAATTATTT | ATGCCTCTCCCTCCT |
| Satt150 | AAGCTTGAGGTTATTCGAAAATGAC | TGCCATCAGGTTGTGTAAGTGT |
| Satt342 | GGTGCAAGGGAAAATGGAAATAA | GATACAACGTCGTGCTACTATCCAAATA |
| Satt373 | TCCGCGAGATAAATTCGTAAAAT | GGCCAGATACCCAAGTTGTACTTGT |
| Satt389 | GCGGCTGGTGTATGGTGAAATCA | GCGCCAAAACCAAAAGTTATATC |
| Satt521 | GCGCTTCACTCTGGTGTAGTGTAG | GCGTTAGATAACGACACATTATTA |
| Satt431 | GCGTGGCACCCTTGATAAATAA | GCGCACGAAAGTTTTTCTGTAACA |
| Satt285 | GCGACATATTGCATTAAAAACATACTT | GCGGACTAATTCTATTTTACACCAACAAC |
| Sat_001 | GCGGATACGACCAAAAATTGTT | GCGAACTGCGAAGATACTACCC |
| Satt641 | GCGGAACATCACGGTTATA | GCGGGAGGCTCTGTCTCTTAGA |
| Sat_330 | GCGTTAGGATTTAGGATGAGGATAGG | GCGCAAATCAGTTGAGCAATGACTTA |
| Satt558 | CTCACACCCTTTCATTATCTA | AAATCGCGCATCTAAATTTAC |
| Satt414 | GCGTATTCCTAGTCACATGCTATTTCA | GCGTCATAATAATGCCTAGAACATAAA |
| Satt102 | CACCTTGCTTCAAAATTC | AATAAGTGAGAGCATAGAAAATAC |
| Satt539 | GCGGTTGTAATTTAATGAACACATT | GCGGATTTTGGACTGGATTAAATAA |
| Satt038 | GGGAATCTTTTTTTCTTTCTATTAAGTT | GGGCATTGAAATGGTTTTAGTCA |
| Satt194 | GGGCCCAACTGATATTTAATTGTAA | GCGCTTTGTGTTCCGATTTTGAT |
| Satt322 | GGGACCCATCATTAAGAA | GTTTTGCCGGACACCACA |
| Satt516 | GCGTTAGCACTATTTTTTTACAAGA | GCGCCGTTCCTCTTTACTTTAT |
| Satt614 | CTCCCCTTTAACCTTTCCTTTATTAG | GCGCGGTAGGAATTAATTGTAGATAGGAT |
| Satt173 | TGCGCCATTTATTCTTCA | AAGCGAAATCACCTCCTCT |
| Sct-189 | CTTTTCCTGGCAATGAT | AAAATCGCAAAACCTTAGT |
| GMES1792 | TGCAAGAAGCAAGTAATCCCT | CCACTTTGCTCTTCTCTGGC |
| aw310961 | GCGCAACTTTTTAGTAAATATTGCATAA | GCGCATACATCTTTTGGGATTTCT |
| Satt154 | AGATACTAACAAGAGGCATAAAACT | AAAGAAACGGAACTAATACTACATT |
| Satt187 | GCGTTTTAATTTATGATATAACCAA | GCGTTTTATCTCTTTTTCCACAAC |
| Satt267 | CCGGTCTGACCTATTCTCAT | CACGGCGTATTTTTATTTTG |
| Satt487 | ATCACGGACCAGTTCATTTGA | TGAACCGCGTATTCTTTTAATCT |
| GMES0002 | TAGGTGACCATGGAGCATCA | CCCCGAAAGAAAGACAGAAA |
| GMES6336 | TTCCCAACAGCTTCAGCTAAC | GGAGGGGCCAAATCTTTATC |
| Satt635 | GCGGTGTATTAAAATTGTCCATGT | GCGCTGTCCTAATTTAAATGAGAAAAC |
| Sat_424 | GCGAATAGAACAAAACAACACTCAT | GCGATGCTGATGCCATTTTCAGAAT |
| Satt478 | CAGCCAAGCAAAAGATAAATAATA | TCCCCCACAAGAGAACAAGAAGGT |
| Sat_272 | GCGATGGCAATATGTTTTTGAGC | GCGGCCTTGTAATTTTCCTTGTTAATGTG |
| Satt022 | GGGGGATCTGATTGTATTTTACCT | CGGGTTTCAAAAAACCATCCTTAC |
| Satt273 | GCGCCTGATTACATTATCGCTTA | GCCTTTCGTTCTCAAACTGAGAGT |
| Satt277 | GGTGGTGGCGGGTTACTATTACT | CCACGCTTCAGTTGATTCTTACA |
| Satt434 | GCGTTCCGATATACTATATAATCCTAAT | GCGGGGTTAGTCTTTTTATTTAACTTAA |
| Satt519 | GGATTTCAAAGAATGAACACAGA | CCGCAAGGTTACGAACTGCTCGAA |
| Satt565 | GCGCCCGGAACTTGTAATAACCTAAT | GCGCTCTCTTATGATGTTCATAATAA |
| Sct_010 | TCCCAAAAGCATTGAG | TATGCACGGAAGAGGA |
| Satt114 | GGGTTATCCTCCCCAATA | ATATGGGATGATAAGGTGAAA |
| Satt588 | GCTGCATATCCACTCTCATTGACT | GAGCCAAAACCAAAGTGAAGAAC |
| Sat_304 | GCGAAACATGGTTGGGTTAAATAATAA | GCGGTCACCGCTAACGAGTTCTTGAA |
| Satt180 | TCGCGTTTGTCAGC | TTGATTGAAACCCAACTA |
| Satt510 | GCGAGTTTCGCCGTTACCACCTCAGCTT | CCCTCTTATTTCACCCTAAGACCTACAA |
| Satt571 | GGGTAGGGGTGGAATATAAG | GCGGGATCCGCGGATGGTCAAAG |
| Satt634 | GCGCATACTGTTTATGCTAGACACCTA | GCGGGGTTGTTTTATTTACAAGAACAG |
| Satt718 | GCGTGCAACACCTCAAGTTTCAAATAC | GCGTAGCTCTTTCCAAAGTTTTCATC |
| Satt453 | GCGGAAAAAAAACAATAAACAACA | TAGTGGGGAAGGGAAGTTACC |
| Satt665 | GCGTTGAATTCGCTGGTTTAATCCTA | GCGCATGACTTAATTTTACACCCTTTATTT |
| AF162283 | GCGAGTTCTGGATGTAGG | GCGTGGCGGCTTTGGTAG |
| Satt130 | TAAACGAAATTTAGTTTTAAGACT | TGAATGGCTAAAAACGTGATT |
| Satt386 | GCGGATGATTTTTATAGAATAGATAAT | CTTCGTTGATACCTCAGTAGAGTACAAA |
| Satt236 | GCGTGCTTCAAACCAACAAACAACTTA | GCGGTTTGCAGTACGTACCTAAAATAGA |
| Satt300 | GCGCCCACACAACCTTTAATCTT | GCGGCGACTGTTAACGTGTC |
| Satt336 | AATTGGAGTGGGTCACAC | TTCCCGGAAAGAAAGAAA |
| Satt070 | TAAAAATTAAAATACTAGAAGACAAC | TGGCATTAGAAAATGATATG |
| Satt243 | GCGCATTGCACATTAGGTTTTCTGTT | GCGGTAAGATCACGCCATTATTTAAGA |
| GMES6346 | TTAATCGTGCCGCTGAATTT | GAATATGATGTGCATGTATTCAAGT |
| Satt600 | GCGCAGGAAAAAAAAACGCTTTTATT | GCGCAATCCACTAGGTGTTAAT |
| Satt286 | GCGGCGTTAATTTATGCCGGAAA | GCGTTTGGTCTAGAATAGTTCTCA |
| Satt146 | AAGGGATCCCTCAACTGACTG | GTGGTGGTGGTGAAAACTATTAGAA |
| Satt409 | CCTTAGACCATGAATGTCTCGAAGATA | CTTAAGGACACGTGGAAGATGACTAC |
| Satt538 | GCAGGCTTATCTTAAGACAAGT | GGGGCGATAAACTAGAACAGGA |
| Sat-099 | GCGAAAATGGCAGAGATAA | AATGCTAAAAGAGGAATGAAATAA |
| Sctt_008 | ACGCAGAGATAGAGAAAGAGA | CCCCCAGACACAACATA |
| Satt390 | AGTGGCTGATAAAAAAAATACTCA | ATAATCGCGGCACAATAATTC |
| Satt650 | CAGTTGGCTGGTCAAATC | TCTGGGTTACTTTTATTGTCA |
| Satt687 | ACCGCAACTCACTCACCTT | GCGCCCAATTAACAGAAAC |
| Satt266 | GCGAAACTCTACGGGTCAGTAGTTAT | GCGTAAGATGGCCTAGAAAGAGGATG |
| Sctt_009 | TGGTAAAGGAGGAACTT | AGAATGTGCTGATGACA |
| GMES5822 | AGGCAGAAACGAAAAAGGGT | AACAACTGGGTGTCCCATTC |
| GMES6735 | CCTTGCTTACACACTTGGCA | TCCAACCTCTACTTTCTGGCA |
| GMES0124 | ATCCAACAGTAGTGCGGCTT | TCGATGATCATTGGGATCAA |
| GMES1173 | TATGGGACATCAAAGCCACA | CGCACTGCCATATGAAGAGA |
| GMES6391 | CGACATCCTCGAAAATCCTC | AAGAGGGAAAGATGGTGGCT |
| GMES0701 | CGAAACGACATCTTTCAGCA | CGTAACCTCTTCGAAGCCTG |
| GMES0963 | AGAGAAGAGAGGAGAGGGGG | AGCGCCTTCACTTCAGACTC |
| GMES3041 | AGCATGGCACTCTAACAGCA | GAAAACAAAGTTTCATCGGGTT |
| GMES5332 | CCCTCTCATTCCTTATGCCA | TCTGTCCCATTTTCCCATGT |
| GMES6776 | GGGACACACATTCCACACAA | TGAGTTTTCCTTGCGTTGC |
| GMES0902 | TTCTATTTCTGGGCGTGACC | CAACAAGGACAAACCCAACC |
| GMES2561 | ATCACGTTTATGCCAAAGCC | ACGCCTTGGGTTTGGTTACT |
| GMES6352 | CGTTCGTCACTCTCCCTCTC | AATTTGTAAGCAGCATGGCA |
| Satt077 | GATCTAAAGTCTGATATTTTTAACTA | AAAAGGAGAAGGAATGC |
| Satt302 | GCGAACTGTAGTTTACTAAAAATAAGTG | GCGGACTGAATTAATATTGGTGTTGAATT |
| Satt334 | GCGTTAAGAATGCATTTATGTTTAGTC | GCGAGTTTTTGGTTGGATTGAGTTG |
| GMES0235 | GAAACTTGGGCAACAGGAAA | AGTTCGCTTCAGACCCAAGA |
| Sat_137 | GATGACGCCGCAGGTTTCTCC | GGTGCGGTTCCACAGTTTTTT |
| GMES4483 | CCGTTGACCATAGTTGGACC | GAGACTGTGCCACCGTGAT |
| GMES4727 | TCAGCCTCGTAACTTGTTTTCA | CATGTGTTTGTGTATTGTACTAGGC |

**Supplementary Table S3.** Ranks of 96 soybean genotypes on AMMI-Estimates for mean seed yield (g/plot) in five environments

| **Code** | **PI Number** | **Ranked on AMMI-Estimates (g/plot)** | | | | | |
| --- | --- | --- | --- | --- | --- | --- | --- |
|  |  | **E1** | **E2** | **E3** | **E4** | | **E5** |
|  |  |  |  |  | |  |  |
| G1 | PI633609 | 92 | 69 | 18 | | 1 | 42 |
| G2 | PI615694 | 69 | 79 | 26 | | 85 | 28 |
| G3 | PI661157 | 19 | 17 | 92 | | 83 | 40 |
| G4 | PI641156 | 86 | 96 | 36 | | 88 | 45 |
| G5 | PI634827 | 85 | 37 | 40 | | 91 | 31 |
| G6 | PI614832 | 14 | 10 | 59 | | 86 | 20 |
| G7 | PI612157 | 81 | 16 | 60 | | 68 | 39 |
| G8 | PI617041 | 53 | 23 | 73 | | 87 | 36 |
| G9 | PI665996 | 40 | 34 | 39 | | 11 | 48 |
| G10 | PI628836 | 62 | 7 | 71 | | 92 | 65 |
| G11 | PI628837 | 89 | 46 | 58 | | 69 | 44 |
| G12 | PI628838 | 67 | 31 | 85 | | 73 | 57 |
| G13 | PI628875 | 20 | 44 | 91 | | 93 | 69 |
| G14 | PI628917 | 42 | 43 | 19 | | 78 | 55 |
| G15 | PI628918 | 56 | 13 | 21 | | 79 | 32 |
| G16 | PI595645 | 60 | 5 | 45 | | 94 | 37 |
| G17 | PI628875 | 78 | 4 | 69 | | 89 | 11 |
| G18 | PI548660 | 61 | 19 | 88 | | 30 | 33 |
| G19 | PI542712 | 80 | 57 | 93 | | 90 | 49 |
| G20 | PI548533 | 28 | 3 | 57 | | 84 | 66 |
| G21 | PI548445 | 87 | 72 | 10 | | 95 | 22 |
| G22 | PI548664 | 15 | 15 | 42 | | 82 | 43 |
| G23 | PI536009 | 68 | 18 | 82 | | 96 | 21 |
| G24 | PI553045 | 16 | 32 | 11 | | 80 | 13 |
| G25 | PI535807 | 82 | 64 | 20 | | 23 | 1 |
| G26 | PI614153 | 94 | 92 | 53 | | 63 | 54 |
| G27 | PI508083 | 35 | 90 | 81 | | 70 | 79 |
| G28 | PI553039 | 37 | 48 | 29 | | 35 | 56 |
| G29 | PI553041 | 43 | 45 | 12 | | 44 | 91 |
| G30 | PI548655 | 93 | 21 | 54 | | 57 | 47 |
| G31 | PI548657 | 76 | 8 | 14 | | 67 | 58 |
| G32 | PI548389 | 74 | 50 | 16 | | 33 | 10 |
| G33 | PI548482 | 25 | 95 | 25 | | 59 | 19 |
| G34 | PI548485 | 47 | 83 | 32 | | 72 | 53 |
| G35 | PI508084 | 24 | 1 | 68 | | 36 | 84 |
| G36 | PI522236 | 58 | 35 | 74 | | 15 | 73 |
| G37 | PI548493 | 96 | 87 | 86 | | 39 | 51 |
| G38 | PI548631 | 71 | 22 | 94 | | 31 | 18 |
| G39 | PI518671 | 88 | 38 | 9 | | 54 | 96 |
| G40 | PI553042 | 7 | 51 | 17 | | 37 | 87 |
| G41 | PI553038 | 52 | 33 | 56 | | 13 | 4 |
| G42 | PI548271 | 26 | 9 | 15 | | 21 | 15 |
| G43 | PI518771 | 65 | 2 | 90 | | 14 | 14 |
| G44 | PI561701 | 91 | 63 | 7 | | 17 | 85 |
| G45 | PI591825 | 49 | 88 | 1 | | 10 | 64 |
| G46 | PI643914 | 46 | 76 | 3 | | 24 | 83 |
| G47 | PI644045 | 59 | 24 | 31 | | 66 | 38 |
| G48 | PI644046 | 84 | 41 | 47 | | 4 | 86 |
| G49 | PI644047 | 29 | 47 | 51 | | 43 | 34 |
| G50 | PI644053 | 55 | 30 | 89 | | 55 | 41 |
| G51 | PI644054 | 57 | 93 | 95 | | 27 | 75 |
| G52 | PI644056 | 17 | 11 | 66 | | 53 | 52 |
| G53 | PI644057 | 22 | 65 | 79 | | 48 | 23 |
| G54 | PI644058 | 30 | 29 | 27 | | 58 | 70 |
| G55 | PI644059 | 54 | 54 | 41 | | 22 | 46 |
| G56 | PI657825 | 27 | 70 | 78 | | 41 | 72 |
| G57 | PI657826 | 6 | 60 | 22 | | 45 | 3 |
| G58 | PI657829 | 1 | 42 | 24 | | 16 | 12 |
| G59 | PI659348 | 41 | 68 | 50 | | 75 | 26 |
| G60 | PI542393 | 31 | 73 | 62 | | 42 | 59 |
| G61 | PI555464 | 90 | 85 | 65 | | 40 | 9 |
| G62 | PI054591 | 70 | 55 | 4 | | 65 | 89 |
| G63 | PI079693 | 23 | 56 | 96 | | 32 | 60 |
| G64 | PI088788 | 8 | 71 | 30 | | 60 | 94 |
| G65 | PI090763 | 12 | 25 | 75 | | 56 | 50 |
| G66 | PI091138 | 45 | 27 | 83 | | 71 | 8 |
| G67 | PI232991 | 39 | 89 | 70 | | 20 | 81 |
| G68 | PI250844 | 77 | 6 | 61 | | 6 | 77 |
| G69 | PI371611 | 36 | 66 | 28 | | 52 | 2 |
| G70 | PI404153 | 32 | 28 | 43 | | 34 | 17 |
| G71 | PI404198A | 48 | 59 | 44 | | 74 | 27 |
| G72 | PI437126A | 64 | 81 | 46 | | 2 | 80 |
| G73 | PI437127B | 33 | 14 | 77 | | 47 | 35 |
| G74 | PI437654 | 9 | 52 | 34 | | 9 | 78 |
| G75 | PI438183 | 2 | 53 | 63 | | 76 | 93 |
| G76 | PI438498 | 51 | 75 | 72 | | 77 | 30 |
| G77 | PI475822A | 72 | 58 | 84 | | 81 | 68 |
| G78 | PI567209B | 95 | 49 | 13 | | 38 | 92 |
| G79 | PI166028 | 4 | 61 | 5 | | 51 | 62 |
| G80 | PI171451 | 13 | 12 | 23 | | 25 | 7 |
| G81 | PI212604 | 34 | 80 | 8 | | 12 | 90 |
| G82 | PI212605 | 44 | 82 | 35 | | 28 | 95 |
| G83 | PI219698 | 38 | 86 | 80 | | 5 | 16 |
| G84 | PI222397 | 21 | 36 | 38 | | 46 | 74 |
| G85 | PI269518C | 73 | 20 | 48 | | 7 | 5 |
| G86 | PI346309 | 5 | 26 | 49 | | 64 | 67 |
| G87 | PI371607 | 63 | 84 | 52 | | 26 | 76 |
| G88 | PI437126C | 10 | 39 | 55 | | 61 | 82 |
| G89 | PI462312 | 79 | 62 | 6 | | 18 | 71 |
| G90 | PI486328 | 50 | 74 | 37 | | 49 | 88 |
| G91 | PI567203 | 75 | 78 | 64 | | 3 | 61 |
| G92 | PI323278 | 66 | 77 | 2 | | 8 | 6 |
| G93 | PNR791 | 83 | 94 | 76 | | 29 | 24 |
| G94 | A6786 | 11 | 40 | 67 | | 50 | 25 |
| G95 |  | 3 | 91 | 87 | | 62 | 29 |
| G96 |  | 18 | 67 | 33 | | 19 | 63 |
